# Single Cell Mechanics in Disease Progression

**DOI:** 10.1002/smsc.70337

**Published:** 2026-07-08

**Authors:** Sabin Kim, Jongmin Lee, Kyungtae Lim, Dong‐Hwee Kim

**Affiliations:** ^1^ KU‐KIST Graduate School of Converging Science and Technology Korea University Seoul Republic of Korea; ^2^ Biomaterials Research Center Biomedical Research Institute Korea Institute of Science and Technology (KIST) Seoul Republic of Korea; ^3^ Department of Life Sciences Korea University Seoul Republic of Korea; ^4^ Department of Integrative Energy Engineering College of Engineering Korea University Seoul Republic of Korea

**Keywords:** cell adhesion, cell junction, cell mechanics, mechanobiology, mechanotransduction, nuclear mechanics

## Abstract

Mechanical force transmission is essential for maintaining cellular structure and function. Forces generated by the extracellular matrix (ECM) and neighboring cells are transmitted via adhesion and junctional complexes to the cytoskeleton and nucleus, forming an integrated mechanical network. These forces at cell–substrate and cell–cell interfaces can be quantified using biophysical tools such as atomic force microscopy, magnetic tweezers, and Förster resonance energy transfer, which have become core technologies for characterizing cellular mechanical properties. Disruption of cellular mechanical homeostasis underlies diverse pathological conditions: In cancer, cell adhesion loss and cytoskeletal reorganization promote metastasis; in fibrosis, increased ECM stiffness enhances cellular contractility; in inflammation, vascular and epithelial barriers are compromised by junctional breakdown; and in cardiomyopathies and skin‐blistering disorders, desmosomal protein defects impair electrical and mechanical coupling. Beyond the cell surface, mechanical forces are transmitted to the nucleus, where they induce lamin–chromatin interactions, lamina‐associated domain rearrangement, and heterochromatin condensation, thereby influencing gene expression and cell fate determination. This review aims to summarize the mechanisms of cellular mechanotransduction from the extracellular microenvironment to the nucleus and discusses related diagnostic and therapeutic strategies. A mechanobiological perspective on disease provides valuable insights for developing treatments for cancer, fibrosis, aging, and laminopathies.

## Graphical abstract

Cells transmit distinct mechanical forces through ECM adhesion and cell–cell junctions and actomyosin‐generated traction forces are transmitted to the substrate through integrin‐based focal adhesions. Mechanical signals are further transmitted to the nucleus via the LINC complex, connecting the cytoskeleton to the nuclear lamina. These forces integrate extracellular and intercellular mechanical cues to regulate nuclear mechanics and mechanotransduction.

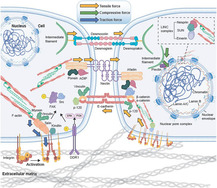

## 1 Introduction

Mechanical forces are key regulators of cellular development, tissue homeostasis, and disease onset [1]. Cells sense external mechanical stimuli and convert them into biochemical signals through mechanotransduction. This mechanochemical coupling is essential for cell proliferation, differentiation, migration, programmed cell death, tissue formation, homeostasis, repair, and pathological processes [2].

Mechanical cues from the extracellular matrix (ECM) and neighboring cells are transmitted to the cytoskeleton and nucleus through focal adhesions (FAs) and adherens junctions (AJs) [1]. FAs are multiprotein complexes comprising integrin receptors and associated signaling and structural proteins that function as mechanosensitive hubs, converting external forces into intracellular signals [3]. As key structures linking the ECM to the actin cytoskeleton, FAs facilitate force transmission, and integrin activation is regulated by force‐sensitive proteins such as talin [4]. Integrins are core components of mature FAs, micrometer‐scale multiprotein complexes that mediate cell–ECM adhesion and mechanical coupling by linking the cytoskeleton to the ECM [5].

Recent studies show that cadherins resist tension and sense and transmit mechanical forces across cell–cell junctions to the actomyosin cytoskeleton, thereby increasing junctional size and complexity [6]. In epithelial cells, AJs sense intercellular tension and function as mechanosensitive complexes that regulate signaling pathways, including the Hippo and Wnt pathways, through E‐cadherin‐mediated mechanotransduction [7].

The cell nucleus maintains its structural integrity and function through interactions among the nuclear envelope, lamins, and chromatin, while also responding to mechanical signals. The inner and outer nuclear membranes (INM and ONM), together with nuclear linkages, transmit forces from the cytoskeleton to the nucleus. These mechanical forces alter nuclear substructure, local chromatin organization, and transcription in a lamin A/C‐dependent manner [8].

Within the nucleus, chromatin and the nucleoskeleton buffer mechanical forces, maintain nuclear elasticity and viscosity, and regulate gene accessibility and transcription through stress‐induced chromatin remodeling. Disruption of these nuclear mechanical properties impairs cell survival and contributes to disease [9].

Dysregulation of mechanotransductive processes is strongly linked to the initiation and progression of multiple diseases. In cancer, reduced cell adhesion and cytoskeletal reorganization promote cell detachment, invasion, and metastasis [10]. In fibrosis, increased ECM stiffness elevates integrin‐mediated tension, creating a positive feedback loop that further enhances cellular contractility and tissue rigidity [11]. In inflammatory diseases, disruption of vascular and epithelial junctions increases permeability, while in cardiomyopathies, defects in desmosomal proteins impair intercellular mechanical coupling [12, 13].

Adhesion‐initiated mechanical signals are also transmitted to the nucleus, inducing lamin–chromatin interactions, lamina‐associated domain (LAD) rearrangement, and heterochromatin condensation alteration, thereby influencing transcriptional regulation and contributing to pathological conditions such as cancer, fibrosis, inflammation, and aging [14].

Therefore, high‐resolution quantitative force measurement tools are essential for understanding adhesion‐dependent mechanical responses. This review aims to summarize current approaches for quantitatively measuring these responses and discuss the diagnostic potential of the resulting mechanical indicators. Reinterpreting cellular and tissue pathology from a mechanical perspective may provide new therapeutic insights into diseases associated with mechanical dysregulation, including cancer, fibrosis, aging, and laminopathies.

## 2 Force Transmission in the Extracellular Microenvironment

### 2.1 Cell Adhesion and Mechanics

#### 2.1.1 Fundamental Principles of Cell Adhesion and Mechanical Interactions

ECM adhesion is essential for maintaining cellular structure and mechanical stability and serves as a key interface for sensing and transmitting mechanical signals [15]. Integrins, the primary ECM adhesion receptors, link the ECM to the actin cytoskeleton, enabling the sensing of mechanical signals and remodeling of the surrounding microenvironment. Through interactions with their ligands, integrins also mediate mechanochemical signal transduction, thereby regulating processes such as cell migration [16, 17].

The cytoplasmic adaptor proteins talin and kindlin bind the β‐integrin cytoplasmic domain and are essential for integrin activation and adhesion. In particular, β_1_‐integrin signaling induces caveolin‐1 phosphorylation via focal adhesion kinase (FAK) and Src kinase, thereby stabilizing lipid raft‐mediated membrane recycling [18]. This stabilization enhances integrin clustering and persistence, thereby promoting FA assembly and actin cytoskeletal reorganization [18]. Ligand‐bound integrins, including those that bind fibronectin or collagen, cluster within the plasma membrane to form nascent adhesions. These adhesions disassemble in response to actomyosin‐generated forces transmitted through talin and vinculin or mature into large, stable FAs [15].

Another ECM adhesion receptor, discoidin domain receptor 1 (DDR1), binds collagen types I, II, and IV [19]. DDR1 enhances collagen adhesion by activating the collagen‐binding integrins α_1_β_1_ and α_2_β_1_ [20]. Upon binding collagen, DDR1 undergoes autophosphorylation at multiple tyrosine residues and activates signaling pathways, including extracellular signal‐regulated kinase, phosphoinositide 3‐kinase (PI3K)/AKT, and Src, thereby regulating cell attachment, migration, proliferation, and survival [20].

**FIGURE 1:**
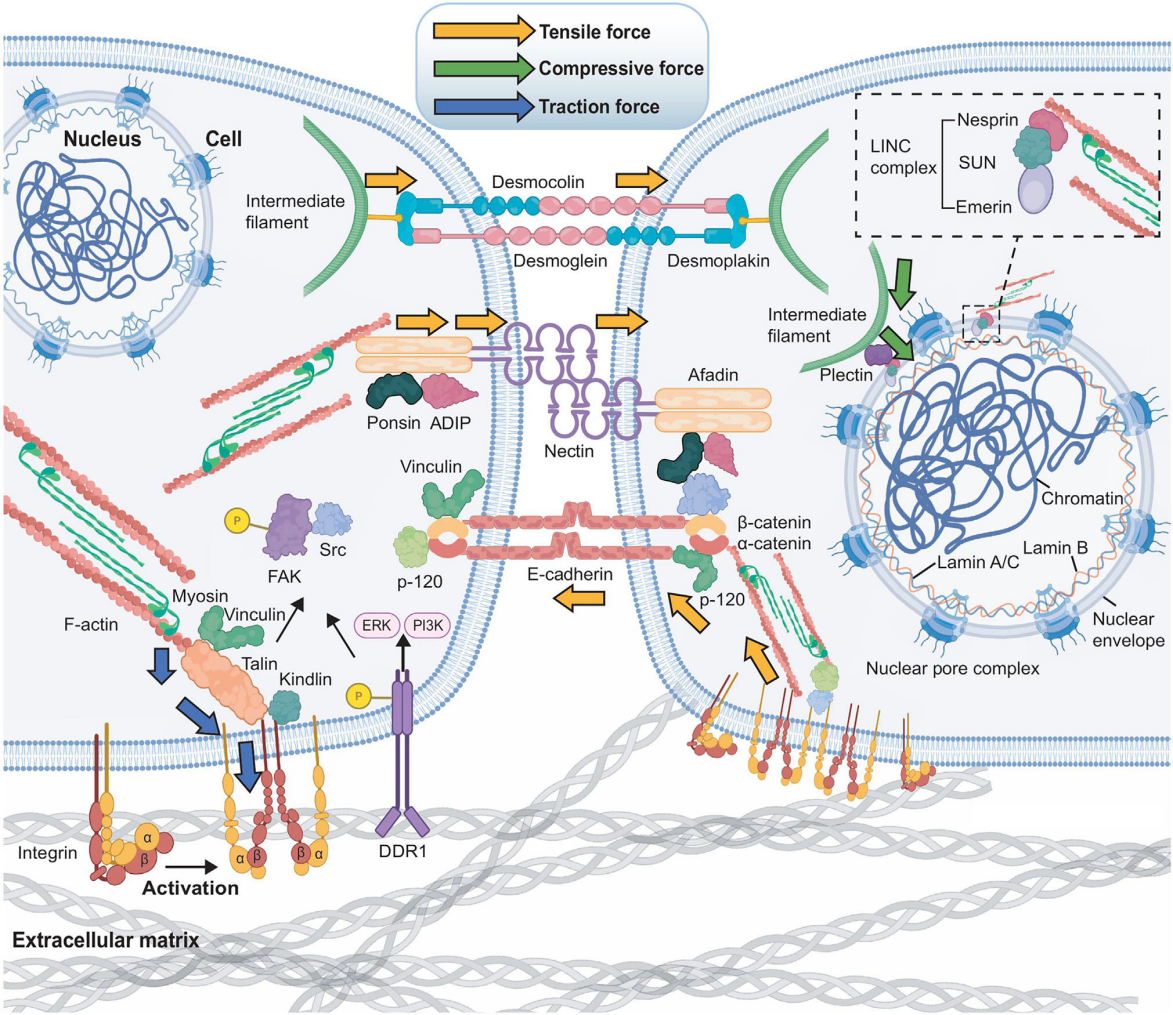
Mechanical forces transmitted from the ECM and cell–cell adhesions to the nucleus. Cells transmit distinct mechanical forces via ECM adhesion and cell–cell junctions. Yellow arrows denote tensile forces, green arrows indicate compressive forces, and blue arrows represent traction forces. At the cell–ECM interface, actomyosin‐generated traction forces are transmitted to the substrate via integrin‐based focal adhesions. These forces are opposed by ECM resistance and transmitted inward through the actin cytoskeleton toward the nucleus. At cell–cell junctions, actomyosin contractility in adjacent cells generates intercellular tensile forces concentrated at adherens junctions, where E‐cadherin complexes mechanically couple neighboring cells. Opposing contractile forces between adjacent cells generate localized compressive stresses in the junctional region and surrounding cytoplasm. Mechanical signals are transmitted to the nucleus via the LINC complex, connecting the cytoskeleton to the nuclear lamina. Actin filaments primarily transmit tensile forces to the nucleus, while intermediate filaments dissipate mechanical loads and provide structural stabilization by buffering compressive and shear stresses. Collectively, these forces integrate extracellular and intercellular mechanical cues to regulate nuclear mechanics and mechanotransduction. ADIP, adipocyte differentiation‐related protein; DDR1, discoidin domain receptor 1; ECM, extracellular matrix; ERK, extracellular signal‐regulated kinase; FAK, focal adhesion kinase; LINC, linker of nucleoskeleton and cytoskeleton; PI3K, phosphoinositide 3‐kinase; SUN, Sad1 and UNC‐84 domain‐containing protein.

During ECM adhesion, cells experience three primary mechanical forces: traction forces exerted on the ECM via integrin‐based adhesions, tensile forces transmitted between neighboring cells through cell–cell junctions, and compressive forces propagated along the cytoskeleton from adhesion‐generated tension at the cell periphery to the nuclear membrane through the linker of nucleoskeleton and cytoskeleton (LINC) complex (Figure 1).

The FA complex is an anchoring and mechanosensitive structure that mediates cell–ECM adhesion and links integrins to actin stress fibers, thereby facilitating force transmission at the cell–ECM interface [21]. FA assembly is a multistep process initiated by integrin binding to the ECM, followed by the recruitment of talin and paxillin and subsequent incorporation of vinculin, α‐actinin, and FAK [22]. The maturation of nascent adhesions into FAs is crucial for cell survival and migration and is driven by FAK‐mediated paxillin phosphorylation, adaptor protein recruitment, and actin filament polymerization [22]. The resulting actin stress fibers, composed of tropomyosin, cross‐linking proteins, and myosin motors, generate forces on adhesions that promote FA maturation and enable cells to sense and transmit mechanical forces [22]. Therefore, actin filaments are central to FA formation and mechanotransduction.

Given that microtubules are dynamic structures that coordinate actin polymerization, cargo transport, and membrane remodeling, they are closely involved in adhesion dynamics and cell migration [23]. Interactions between microtubules and integrin‐mediated adhesion complexes modulate myosin IIA filament assembly by regulating Rho/ROCK signaling [24]. Consequently, these myosin IIA filaments act as effectors that regulate integrin‐mediated adhesion [24].

Intermediate filaments stabilize cellular architecture by distributing and modulating mechanical forces transmitted from the ECM, thereby supporting cell adhesion and migration [25]. These filaments also interact directly with FA proteins to regulate adhesion dynamics [21]. Vimentin, a major intermediate filament protein, extends throughout the cytoplasm and into cellular protrusions, where it forms an interpenetrating network with the actin cytoskeleton that provides mechanical support for FAs [25]. These interactions are strongly influenced by ECM mechanical properties, including stiffness and density. Moreover, vimentin enhances stress fiber formation and local adhesion maturation, increasing cellular traction forces [16, 25].

#### 2.1.2 Biophysical Techniques and Pathological Applications for Analyzing Cell Adhesion

Cells perform key physiological functions, including survival, differentiation, and migration, and regulate signaling through interactions with their microenvironment [26]. Understanding the physical and functional properties of cell–cell and cell–microenvironment interactions is essential for characterizing cellular behavior under normal and pathological conditions [26].

Since these interactions are regulated by biochemical signals and mechanical forces, diverse single‐cell measurement techniques, including atomic force microscopy (AFM), tension gauge tether (TGT), micropipette aspiration, traction force microscopy (TFM), magnetic tweezers (MTs), and optical tweezers (OT), have been developed to quantitatively assess distinct aspects of cellular mechanics (Table 1). These techniques provide complementary information across multiple spatial and force scales.

**TABLE 1:** Experimental force‐based techniques for measuring cellular, nuclear, and chromatin mechanics.

Technique	Purpose	Measurement principle	Key analysis parameters	References
RWG	High‐throughput quantification of single‐cell adhesion force and energy	RGD‐functionalized RWG sensors detect adhesion‐induced shifts in resonant wavelength, and force–distance curves are used to quantify adhesion force and energy	WS, IWS, adhesion force (nN), adhesion energy (J), F–Z curve	[27]
TGT	Determination of force thresholds required for receptor‐mediated adhesion	DNA duplexes or hairpins are designed to rupture when subjected to forces exceeding predefined thresholds	Rupture threshold T_tol (pN), receptor specificity	[28]
MTFM	Simultaneous quantification of molecular‐scale integrin forces and whole‐cell traction forces	Gold nanoparticle‐conjugated DNA tension probes report integrin forces via fluorescence changes, while embedded fluorescent beads enable TFM measurements of cell‐generated forces	Molecular tension fluorescence signal, DNA force threshold (pN), integrin force distribution, bead displacement, traction stress, hydrogel stiffness	[29]
RAD‐TGT	High‐throughput cell mechanotyping of cells	Ligands tethered to force‐sensitive DNA constructs rupture above defined force thresholds. Mechanical forces are encoded as intracellular fluorescence or DNA barcode signals and quantified using flow cytometry or sequencing	T_tol (pN), tension per cell	[30]
FluidFM	Independent control of indentation and suction for probing cellular mechanical responses	A microfluidic cantilever applies independently controlled indentation and suction pressures, while cantilever deflection provides quantitative force measurements in living cells	Indentation force (nN), suction pressure (mbar), cantilever deflection, F–Z curve, membrane tension	[31]
TFM	Quantification of cell–substrate traction forces and spatial force distribution	Cell–substrate traction forces are quantified from substrate bead displacements measured before and after detachment	Bead displacement (µm), tensile stress (Pa), tensile force vector, substrate stiffness (kPa)	[6]
FRET	Dynamic detection of molecular tension and protein conformational changes	Fluorescent donor–acceptor pairs detect changes in intermolecular distance or force‐induced structural rearrangements	FRET efficiency/lifetime, permissible tension (pN)	[32]
Nanoneedle AFM	Direct measurement of nuclear mechanical properties	Nanoneedle indentation combined with force–distance analysis and elastic modeling enables spatial mapping of nuclear stiffness	Indentation force (nN), indentation depth (nm), F–z curve, Young's modulus, elastic map	[33]
Micropipette aspiration	Quantification of nuclear deformation and viscoelastic properties	Negative pressure aspirates the nucleus into a micropipette, and protrusion length is used to evaluate mechanical properties	Suction pressure (mbar), estimated nuclear force (nN), nuclear strain	[34]
MT	Single‐molecule force and torque measurements of nucleic acids and chromatin	External magnetic fields manipulate superparamagnetic beads to apply controlled forces and torques	Force (pN), torque, elastic modulus, viscosity coefficient, stiffness increase ratio, curvature, extension (nm–µm)	[35]
OT	Analysis of force‐dependent chromatin and nucleosome dynamics at the single‐molecule level	Focused laser beam traps dielectric beads attached to nucleic acids or chromatin and measures force‐dependent structural changes using optical manipulation	Force (pN), DNA extension (nm)	[36]

Abbreviations: AFM, atomic force microscopy; DNA, deoxyribonucleic acid; FluidFM, fluid force microscopy; FRET, Förster resonance energy transfer; IWS, integrated wavelength shift; J, joule; kPa, kilopascal; mbar, millibar; MT, magnetic tweezers; MTFM, molecular tension fluorescence microscopy; nm, Nanometer; nN, nanonewton; OT, optical tweezers; Pa, pascal; pN, piconewton; RAD‐TGT, rupture and deliver tension gauge tether; RGD, arginine–glycine–aspartic acid; RWG, resonant waveguide grating; T_tol, rupture threshold force; TFM, traction force microscopy; TGT, tension gauge tether; WS, wavelength shift.

AFM enables high‐resolution quantification of cell stiffness and adhesion by measuring cantilever deflection in response to forces at the cell surface [37]. AFM‐applied shear forces can also be used to isolate cells and assess cell–substrate adhesion [38]. Cantilevers are highly sensitive and deform in response to minute forces generated when the probe contacts or approaches the surface [39]. Their deflection is typically monitored using laser‐based detection systems in which laser beams reflected from the cantilever are projected onto a detector, converting nanoscale displacements into measurable electrical signals [39]. AFM‐derived mechanical parameters are commonly estimated using contact mechanics models, such as the Hertz model, to calculate material properties, including Young's modulus (E), although these estimates may vary depending on assumptions regarding cell geometry and viscoelasticity [39].

Forces exerted on individual receptor–ligand bonds typically fall within the piconewton (pN) range, which requires highly sensitive molecular‐scale force sensors such as TGT. TGT is a molecular force sensor that uses the rupture force of double‐stranded DNA (dsDNA) to determine whether receptor‐transmitted forces exceed predefined tension thresholds [40, 41]. In this system, the dsDNA tether remains stable up to a defined force threshold. When forces transmitted through receptor–ligand interactions exceed this threshold, the dsDNA irreversibly dissociates, generating a fluorescent signal that indicates force‐induced events [42]. Therefore, TGT provides a digital readout of whether the applied force exceeds a defined threshold, enabling precise measurement of the maximum tension across individual molecular interactions [42]. These features make TGT particularly useful for investigating force‐dependent receptor signaling in cell–matrix interactions, cell–cell adhesion, mechanosensitive signaling, and tumor‐associated matrix remodeling at the molecular level [43].

Micropipette aspiration evaluates cellular and nuclear mechanics by applying controlled suction through a fine pipette positioned on the cell or nuclear surface [26]. The micropipette forms a seal with the nucleus of a living adherent cell (or an isolated nucleus), and suction generates tensile forces at the nuclear surface [26]. Translating the micropipette deforms the nucleus while enabling real‐time imaging of its response.

TFM is widely used to quantify cellular traction forces exerted on compliant substrates, enabling the reconstruction of spatial stress maps within soft elastic gels [6]. In this technique, cells are cultured on elastic substrates containing reference markers, including fine fluorescent beads, which are displaced when cells exert force [6]. Bead displacement is used to quantify the magnitude and direction of cellular traction forces [6]. Traction fields are then reconstructed from displacement fields using mechanical models of the elastic substrate and computational methods, including the finite element, boundary element, and Fourier‐space methods [44].

MTs evaluate cellular and nuclear mechanics by applying forces to superparamagnetic beads using external magnetic fields [45]. Magnetic field gradients generated by a current‐carrying coil and soft magnetic core exert forces on the beads, and bead displacement is converted into force using Stokes’ law [45]. This approach enables estimation of the relationship among applied current, distance, and force. MTs provide precise nanonewton (nN)‐scale force application for simulating physiological cellular responses. These techniques enable quantitative measurement of adhesion and mechanical properties at the single‐cell level and can be selected according to specific research objectives based on their principles, strengths, and limitations. They are essential for quantitatively analyzing differences in adhesion between normal and tumor cells, cell–matrix interactions, and mechanosensitive cellular behaviors.

Additionally, OT uses focused lasers to manipulate single cells without direct physical contact, thereby minimizing mechanical perturbation and preserving cellular integrity during measurement [26]. OT is particularly well suited for probing local membrane viscoelasticity at low forces (0.1–100 pN), and adjusting laser wavelength and power appropriately minimizes photodamage during long‐term experiments. Multiple probes can simultaneously manipulate and track different cellular regions, while force protocols—including single‐pulse, repetitive‐pulse, and step‐type protocols—enable analysis of elasticity, viscosity, nonlinear stress responses, and fluidization [45].

In cancer tissues, cell–substrate interactions are critical for tumor development and progression. β_5_ integrin subunits, whose expression is regulated by transforming growth factor‐β (TGF‐β) signaling, pair with α_v_ subunits to form α_v_β_5_ integrins that recognize arginine‐glycine‐aspartic acid (RGD) motifs and induce various cellular responses [10].

During tumor progression, α_v_β_5_ integrin activates the FAK–steroid receptor coactivator pathway, regulates cell migration and matrix remodeling, and complements α_v_β_3_ integrin‐mediated p21‐activated kinase signaling [10]. In pancreatic ductal adenocarcinoma, cancer‐associated fibroblasts (CAFs) and TGF‐β secreted by epithelial cancer cells upregulate β_5_ integrin expression, thereby enhancing intercellular adhesion and invasive capacity [46]. Overall, changes in β_5_ integrin‐mediated forces reinforce tumor cell mechanical properties [46].

**FIGURE 2:**
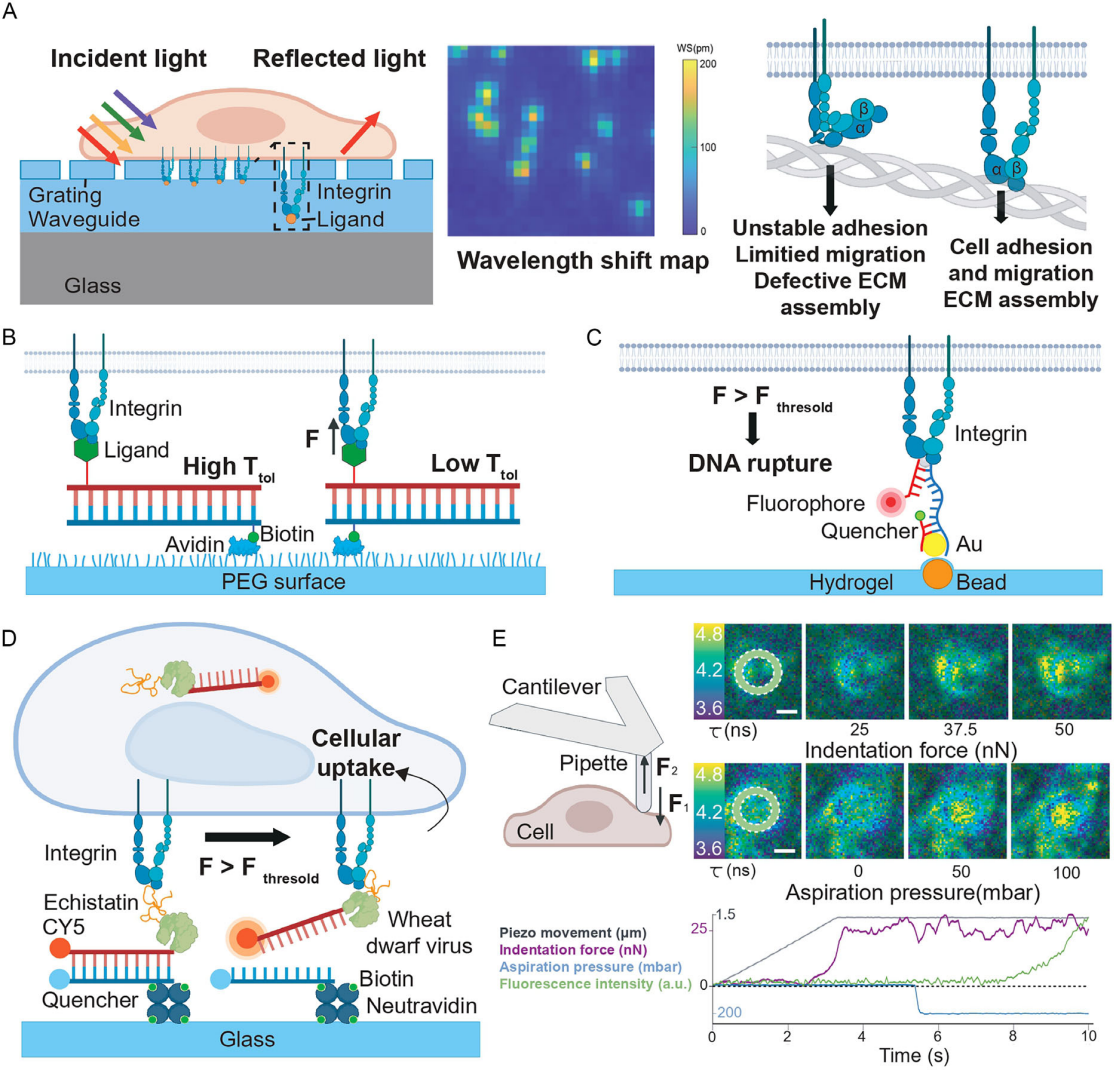
Quantification of cell–substrate adhesion and force generation across molecular and cellular scales. (A) Conceptual schematic of an RWG sensor. The RWG sensor couples incident light at a specific wavelength into a guided wave mode via a periodic diffraction grating on the waveguide surface. At resonance, an evanescent field (∼150 nm penetration depth) forms above the waveguide surface and responds sensitively to local refractive index changes near the sensor interface. Cell adhesion via integrin–RGD interactions alters the effective refractive index, producing a measurable shift in resonant wavelength. This optical shift enables high‐resolution readout of adhesion strength, where yellow indicates strong adhesion and blue denotes weak adhesion. Adapted with permission [27] 2021, *Scientific Reports*. (B) Conceptual schematic of TGT. Mechanosensitive DNA constructs activate when applied tension exceeds a defined rupture threshold. Double‐stranded DNA serves as a force‐sensitive tether; ligand binding to integrins applies mechanical load to the DNA linker. Depending on force magnitude, the tether ruptures or remains intact, allowing indirect quantification of cellular traction forces. Adapted with permission [28] 2013,*Science*. (C) Conceptual schematic of modified mTFM on soft hydrogels. DNA‐based tension probes are densely conjugated to Au NPs and immobilized on hydrogel surfaces via lipoic acid–PEG linkers, forming stable Au–thiol bonds that confine probes to the surface and reduce background signal. Integrin binding to ligand‐functionalized DNA probes generates mechanical tension that deforms the probes, causing fluorescence changes that report molecular‐level integrin forces. Simultaneously, embedded fluorescent nanobeads track hydrogen deformation induced by cellular traction forces, enabling mapping of cell‐scale force distributions. Adapted with permission [29] 2023, *Nature Methods*. (D) Conceptual schematic of RAD‐TGTs. Cells adhered to RAD‐TGT surfaces generate mechanical tension through integrin engagement. Integrins couple to DNA probes via an Echi–WDV linker, forming an integrin–Echi–WDV–DNA mechanical linkage. When the applied force exceeds the rupture threshold, DNA duplexes dissociate and release fluorescently labeled ligands (Cy5) or DNA barcodes internalized by cells. Flow cytometry quantifies intracellular signals across large cell populations, generating mechanotype profiles that encode cumulative force history at the single‐cell level. Adapted with permission [30] 2023, *Nature Communications*. (E) Conceptual schematic of a force‐controlled nanopipette. This system allows independent control of indentation force (nanonewton scale) via cantilever deformation and aspiration pressure (millibar scale) via pneumatic suction. Changes in membrane tension induced by mechanical stimulation are quantified using FLIM. In FLIM, light green circles indicate the pipette tip, and a blue‐to‐yellow shift denotes increasing membrane tension. Increased membrane tension activates mechanosensitive ion channels, producing calcium‐dependent fluorescent signals. Adapted with permission [31] 2024, *Nature Methods*. Au NPs, gold nanoparticles; Cy5, cyanine 5; Echi–WDV, echistatin‐wheat dwarf virus; FLIM, fluorescence lifetime imaging microscopy; mTFM, molecular tension fluorescence microscopy; RAD‐TGTs, rupture and deliver tension gauge tethers; RGD, arginine–glycine–aspartic acid; RWG, resonant waveguide grating; TGT, tension gauge tether; PEG, polyethylene glycol.

To quantitatively assess these adhesion‐mediated mechanical changes, resonant waveguide grating (RWG) biosensors use a high‐refractive‐index waveguide layer and fine diffraction grating to detect changes in the surface refractive index via wavelength shifts (WS) (Figure 2A) [27]. The grating compensates for the momentum mismatch between incident light in free space and the waveguide mode, allowing light to couple into the waveguide only at specific wavelengths, thereby forming a resonant state [47]. This resonant mode generates an evanescent field that extends hundreds of nanometers from the waveguide surface and is highly sensitive to changes in mass redistribution and the refractive index of attached cells [27].

Poly‐L‐lysine‐graft‐poly(ethylene glycol) (PLL‐g‐PEG) coatings containing RGD motifs selectively bind αIIbβ_3_, α_v_β_3_, and α_5_β_1_ integrins on HeLa cells, promoting initial adhesion and increasing local refractive index changes and WS during cell spreading and attachment [27]. The WS image is converted into an integrated wavelength shift (IWS) by summing pixels above a defined threshold, providing a quantitative proxy for cell contact mass and adhesion progression (Figure 2A) [27].

Maximum adhesion force and adhesion energy are then derived from force–distance curves generated by mechanically detaching the same cell using a fluidic force microscopy (FluidFM) BOT system [27]. Linear regression analysis of IWS and these measurements produces a correction formula that converts IWS into corresponding mechanical parameters [27]. By combining the label‐free, high‐throughput capabilities of RWG with the single‐cell precision of FluidFM, this integrated approach enables quantitative assessment of real‐time adhesion dynamics and adhesion energy in large cell populations [27].

Analysis of HeLa Fucci2 cells reveals a linear increase in basal adhesion area from the mitotic (M)/gap 1 (G1) transition through the G1 phase, whereas total cell surface area continues to increase until the late synthesis (S)/gap 2 (G2) phase [27]. Integrin‐mediated forces increase from the M/G1 transition to the G1 phase, remain elevated through early S phase, and decrease slightly during the late S/G2 phase [27]. In M‐phase cells, resistance to hydrodynamic lift is significantly lower when measured using robotic FluidFM, indicating that integrin‐mediated adhesion and cytoskeletal connectivity vary with cell‐cycle stage and cell morphology [27]. This approach enables real‐time quantification of cancer cell mechanics in relation to cytoskeletal organization, integrin expression, and cell‐cycle stage. HeLa cells exhibit pronounced cell cycle‐dependent differences in adhesion and integrin‐mediated forces [39]. Cell surface area and adhesion are reduced during the M and late G2 phases but remain elevated during the G1 and S phases [48].

TGT is a nanoscale sensor that defines the maximum tension tolerance (T_tol) that cell–ligand bonds can sustain, enabling quantitative determination of molecular tension thresholds during ligand engagement (Figure 2B). The system uses a DNA double helix as the tether and RGDfk as the ligand for integrin α_v_β_3_ binding [40]. DNA tethers with varying T_tol values are designed based on differences in rupture force, unzipping geometry, and shear geometry [28]. Using TGT, the molecular tension produced by integrin α_v_β_3_ in cancer cells can be analyzed, enabling the exploration of mechanics‐based cancer diagnostics.

On DNA‐tethered surfaces, T_tol ≤ 33 pN nearly abolishes cell adhesion, whereas T_tol ≥ 43 pN supports substantial adhesion, with the number of adherent cells gradually increasing as T_tol increases [28]. These findings indicate that the T_tol generated by α_v_β_3_ binding a single RGDfK ligand ranges from 33 to 43 pN [28]. Reducing ligand density on TGT surfaces did not alter this threshold, which is consistently observed across multiple cell lines, including MDA‐MB‐231, HeLa, HEK293, and NIH3T3 cells, suggesting a conserved molecular tension threshold for α_v_β_3_ integrin activation [28]. These findings suggest that cancer cells exert a minimum mechanical force during early adhesion.

Under hyperosmotic conditions that reduce membrane tension, the threshold decreases to 23–33 pN, whereas blebbistatin treatment (0–100 μM) has no detectable effect [28]. This suggests that α_v_β_3_‐mediated molecular tension during initial adhesion is primarily regulated by membrane tension. Regarding FA formation, CHO‐K1 cells adhere within 30 min on surfaces with T_tol values of 43 or 56 pN, although FAs are not observed at this stage. After 1–2 h, FAs and stress fibers form only on the 56 pN surface [28]. These findings suggest that approximately 40 pN is sufficient for initial adhesion, whereas higher tension is required for FA maturation. Identifying a molecular tension threshold of ≥56 pN provides a mechanical criterion for distinguishing between initial and stable adhesion [28]. This tension‐based distinction establishes a quantitative framework for evaluating cancer cell adhesion and mechanosensitivity and may serve as a novel diagnostic indicator of cell–matrix interactions.

Fibrosis is associated with tissue stiffening resulting from excessive ECM accumulation. Mechanosensitive pathways, including TGF‐β and yes‐associated protein (YAP)/transcriptional coactivator with PDZ‐binding motif (TAZ) signaling, promote cellular activation, while the stiffened microenvironment further enhances cell contractility and ECM synthesis, forming a mechanical feedback loop [49]. Fibroblasts normally maintain tissue homeostasis, but mechanical stimuli and soluble factors enhance ECM production and contractility, thereby contributing to pathological fibrosis [11]. This process is essential for wound healing and development, but also contributes to chronic disease progression.

In late‐stage fibrosis, cells sense ECM rigidity and deformation, further increasing actomyosin contractility [11]. Key pathological features of fibrosis include tissue stiffening and increased cell contractility; therefore, quantifying forces at the cellular and tissue levels may provide an important indicator of early diagnosis and disease progression. Increased ECM stiffness promotes fibroblast mechanotransduction and is associated with enhanced contractility, stress fiber formation, and elevated α‐smooth muscle actin (α‐SMA) expression [11]. Therefore, techniques that quantitatively measure cellular traction forces in response to substrate stiffness are essential for the early diagnosis and monitoring of fibrosis progression.

Using molecular TFM (mTFM), DNA tension probes are selectively immobilized on hydrogel surfaces, enabling real‐time imaging of pN‐scale forces transmitted from cells to the ECM through integrins [29]. Conventional mTFM is initially limited to rigid substrates because porous, heterogeneous hydrogels absorb molecular tension probes, resulting in high background signals. To overcome this limitation, advanced mTFM platforms use hydrogels with tunable stiffness ranging from 1 to 80 kPa and functionalize them with DNA–gold nanoparticle‐based tension probes (Figure 2C) [29]. In these systems, gold nanoparticles serve as docking sites for probe immobilization, and DNA tension probes are covalently attached via COOH‐PEG8‐SH linkers. [29].The large size of these complexes prevents their penetration into the hydrogel matrix, thereby markedly reducing background signals and confining the probes to the hydrogel surface [29]. Conversely, fluorescent nanobeads enable TFM to measure whole‐cell traction forces, whereas mTFM simultaneously quantifies forces generated through specific molecular interactions [29]. When force is transmitted through integrin receptors bound to DNA probes on the hydrogel surface, probe deformation generates corresponding fluorescence changes (Figure 2C). These fluorescence changes are then quantified to determine the forces transmitted through individual integrins.

Fibroblasts cultured on these substrates exhibit greater cell spreading and larger FAs as ECM stiffness increases, whereas the average tension transmitted per integrin molecule remains unchanged [29]. Furthermore, a locking‐strand assay with mTFM reveals that the kinetics of integrin–ECM coupling vary with ECM stiffness. On soft hydrogels (1 kPa), cells repeatedly attach and detach while probing the substrates, whereas stable FA formation predominates at intermediate stiffness (6–30 kPa) [29]. However, at high stiffness (>80 kPa), adhesion–detachment cycling increases again, suggesting that excessive ECM rigidity, observed in fibrotic tissues, promotes cell adhesion instability and aberrant cytodynamics [29]. Collectively, the quantitative assessment of integrin tension and coupling dynamics using mTFM may provide a mechanobiological approach for evaluating fibrosis‐associated changes in ECM stiffness and cellular force transmission.

Inflammation alters biochemical signaling, structural integrity, and mechanical coupling networks. During inflammatory responses, changes in ECM composition and stiffness are accompanied by alterations in ECM receptor expression and activation. These inflammation‐induced changes remodel the ECM and activate integrins, thereby enhancing cell adhesion and initiating outside‐in signaling that further amplifies mechanical and inflammatory responses. This process creates conditions that enable leukocytes to selectively arrest and adhere to blood vessels near inflammatory sites. Mechanical tension generated through integrin–ligand interactions may further stabilize adhesion and amplify inflammatory signaling during inflammation [50]. Traction forces at integrin binding sites enhance bond stability and, under certain conditions, induce force‐dependent behavior in which bond lifetime and strength vary with the applied force [51]. Therefore, understanding the mechanisms underlying integrin activation may provide a theoretical basis for developing therapies that selectively modulate inflammatory responses.

DDR1, a collagen‐activated atypical receptor tyrosine kinase, enables cells to sense ECM physical properties and coordinately regulate the cytoskeleton and adhesive complexes [12]. Under inflammatory conditions, increased collagen reorganization and oxidative ECM modifications alter DDR1 recognition and activity [12]. In some cell types, DDR1 hyperactivation promotes excessive contractility via the RhoA/ROCK pathway, thereby disrupting tension homeostasis and impairing cell–cell connectivity and barrier function [12]. Conversely, interactions between DDR1 and E‐cadherin signaling help maintain mechanical balance across cytoskeletal, junctional, and nuclear networks during inflammation.

Rupture‐ and delivery‐based TGTs (RAD‐TGTs) extend conventional TGT technology by converting cellular mechanical forces into intracellularly retained DNA rupture events, thereby enabling high‐throughput, single‐cell quantification of mechanical history (Figure 2D) [30]. When forces transmitted through ligand binding exceed a defined rupture threshold, an irreversible molecular record is generated. RAD‐TGTs consist of DNA double helices engineered to rupture at predetermined force thresholds [30]. The rupture force of a DNA double helix is determined by its GC base‐pair content and binding geometry, which establish the minimum cellular force required for rupture [30]. Based on this principle, TGTs with rupture thresholds of approximately 12 pN and 54 pN are used to distinguish cellular mechanical behaviors across different force regimes [30].

One DNA strand is immobilized on the substrate via biotin–streptavidin or neutravidin interactions, while the complementary strand presents a ligand that binds cell‐surface receptors (Figure 2D) [30]. To facilitate ligand conjugation, the Wheat Dwarf Virus (WDV)‐derived HUH tag specifically recognizes single‐stranded DNA, enabling efficient, site‐specific covalent attachment of protein ligands to DNA probes. This strategy facilitates the straightforward conjugation of integrin‐binding ligands, including fibronectin domains and echistatin, a high‐affinity ligand that binds multiple integrins to promote cell adhesion and force transmission [30]. When cells are cultured on RAD‐TGT‐coated surfaces, they bind the ligand‐bearing strand through adhesion receptors such as integrins and transmit actomyosin‐generated forces to the substrate. When these forces exceed the DNA rupture threshold, the double helix ruptures, resulting in internalization of the ligand strand. RAD‐TGTs enable high‐throughput analysis of these rupture events using two complementary strategies [30]. First, Cy5‐labeled ligand strands accumulate within cells and can be quantified via flow cytometry, enabling rapid profiling of the mechanical states of tens of thousands of cells [30]. Cellular fluorescence intensity reflects the cumulative mechanical forces exerted over a defined period. Second, ligand strands containing unique DNA barcodes instead of fluorophores enable the simultaneous use of multiple RAD‐TGT types [30]. The internalized barcodes are subsequently quantified using PCR amplification and next‐generation sequencing, enabling multiplexed single‐cell analysis of force transmission across different rupture thresholds and ligand conditions [30].

Using a WDV–echistatin ligand, U251 glioblastoma cells exhibit significantly higher RAD‐TGT rupture signals than those of CHO‐K1 cells. Under 12 pN RAD‐TGT conditions, U251 cells show an approximately 12‐fold increase in fluorescence intensity relative to that of baseline, whereas CHO‐K1 cells show an approximately sevenfold increase [30]. This disparity persists under 54 pN RAD‐TGT conditions, with U251 cells showing an approximately sevenfold increase in fluorescence intensity compared with an approximately threefold increase in CHO‐K1 cells [30]. These findings indicate that U251 cells generate stronger integrin‐mediated tension under low‐ and high‐threshold force conditions. This behavior may reflect a mechanical phenotype associated with integrin hyperactivation in inflammatory or tumor microenvironments.

Analysis of RAD‐TGT rupture in Talin‐1‐deficient U251 cells provides insight into the molecular basis of this enhanced force transmission. Under WDV–echistatin conditions, wild‐type (WT) U251 cells exhibit an approximately 2.5‐fold increase in fluorescence intensity relative to that of baseline under 12 pN RAD‐TGT conditions [30]. In contrast, Talin‐1 knockout (KO) U251 cells show a 25%–30% reduction in RAD‐TGT rupture‐associated fluorescence compared with that of WT cells under identical conditions [30]. This role of Talin‐1 is further supported under high‐threshold (54 pN) RAD‐TGT conditions, where WT U251 cells maintain high fluorescence responses, whereas Talin‐1 KO cells exhibit a 35% reduction in RAD‐TGT rupture [30]. This indicates that Talin‐1 contributes to low‐threshold force transmission and functions as a key mechanical coupler linking integrins to the actomyosin cytoskeleton, thereby facilitating force transmission across a wide tension range. The differences in RAD‐TGT rupture observed between U251 and CHO‐K1 cells under WDV–echistatin conditions likely reflect distinct integrin activation states and mechanical phenotypes [30]. Furthermore, Talin‐1 deficiency reduces RAD‐TGT rupture by approximately 25%–30% at 12 pN and 35% at 54 pN, suggesting that integrin‐mediated tension transmission depends strongly on Talin‐1 across force scales [30]. Therefore, understanding integrin activation mechanisms may offer a theoretical basis for developing therapies that selectively modulate inflammatory responses.

Using the Membrane Tension Sensor System, a Förster resonance energy transfer (FRET)‐based membrane tension biosensor, real‐time imaging reveals that laminar shear stress decreases the FRET ratio in endothelial cells, indicating increased membrane tension accompanied by DDR1 condensate formation [12]. These condensates gradually disappear when cells return to static conditions, suggesting that DDR1 responses to membrane tension are reversible [12]. To investigate the structural mechanosensitivity of DDR1, the unfolding dynamics of its ectodomain are analyzed using MTs. The purified DDR1 ectodomain is prepared by attaching a DNA strand to one terminus and biotin to the other, enabling its anchoring to surface‐immobilized antibodies and streptavidin‐coated magnetic beads [12]. Applying forces of 1–30 pN through a magnetic field allows real‐time measurement of DNA extension, and the resulting force–extension curves are used to characterize DDR1 folding and unfolding dynamics [12]. Measuring DDR1 height under constant forces of 1–30 pN reveals a distinct unfolding event at approximately 20.7 pN [12]. However, after 5 min of shear‐flow exposure, the unfolding force of the same DDR1 molecule reduces to approximately 5.5 pN [12]. These findings suggest that shear stress alters DDR1 conformational stability and reduces its resistance to mechanical unfolding [12]. Therefore, DDR1‐mediated mechanosensing in endothelial cells represents a distinct force‐dependent pathway that links shear stress to inflammatory signaling. This pathway complements integrin‐based mechanotransduction and highlights the diverse mechanical inputs that shape inflammatory responses.

Angiogenesis—the formation of new blood vessels from preexisting ones—is essential for tissue regeneration and wound healing [52]. Mechanical forces, including shear stress, cyclic stretch, and intraluminal pressure, shape vascular structure and patterning by regulating endothelial cell sprouting, migration, lumen formation, and polarity. Mechanical regulation is crucial during wound healing. Biomechanical forces generated during tissue contraction and ECM remodeling are transmitted to endothelial cells, stimulating neovascularization and promoting tissue regeneration [52]. Additionally, external mechanical stimuli influence vascular morphology and angiogenesis, thereby affecting wound healing [53]. During angiogenesis, endothelial tip cells form actin‐based protrusions, including lamellipodia and filopodia, that guide directional migration [53]. The formation and dynamics of these structures are regulated by actin‐related protein 2/3 (Arp2/3) complexes and formin proteins, which are essential for cell migration and junction remodeling [53]. Therefore, angiogenesis during wound healing depends not only on chemical signals but also on mechanical forces, including membrane tension, intracellular pressure, and actin‐mediated polarity. Piezo1 and Piezo2, members of the Piezo protein family, are mechanically activated cation channels that form homooligomeric complexes [54]. They function as molecular mechanosensors that generate ion currents in response to external mechanical stimuli. In endothelial cells, Piezo1 is directly activated by fluid shear stress [54]. Global or endothelial‐specific Piezo1 deficiency causes severe defects in vascular development and remodeling, highlighting its essential role in flow sensing and vascular structure formation and maintenance [54]. Piezo1 is widely recognized as a mechanosensor of forces, including membrane tension and shear stress, thereby contributing to Ca^2+^ influx and downstream mechanosignaling pathways [55]. Therefore, understanding angiogenesis, vascular remodeling, and tension and blood pressure regulation requires approaches that quantitatively link changes in membrane tension, a key regulator of Piezo1 activity, to Piezo1‐mediated mechanosignaling.

The force‐controlled nanopipette integrates a microfluidic channel within the cantilever, enabling precise and independent control of membrane indentation force and aspiration pressure (Figure 2E) [31]. While nN‐scale indentation forces are maintained, internal probe pressure, measured in millibars (mbar), is used to aspirate the cell membrane [31]. Membrane tension is quantitatively measured using the fluorescence‐based Flipper‐TR probe, which consists of a fluorescent emitter and a flipper moiety embedded within the lipid bilayer [31]. The rotational freedom of the flipper moiety depends on the physical properties of the surrounding membrane. Low membrane tension permits rotation of the flipper moiety, reducing fluorescence lifetime, while high tension restricts rotation and increases fluorescence lifetime [31]. Consequently, Flipper‐TR fluorescence lifetime correlates linearly with local changes in membrane tension. This approach enables high‐resolution spatiotemporal mapping of membrane tension using fluorescence lifetime imaging microscopy (Figure 2E) [31]. Experiments combining FluidFM and Flipper‐TR directly assess how mechanical stimulation of the cell membrane alters tension and activates the mechanosensitive ion channel Piezo1. Indentation of the cell membrane with varying forces (e.g., 25–50 nN) using the FluidFM tip increases local membrane tension, as indicated by changes in the Flipper‐TR fluorescence lifetime (Figure 2E) [31]. This finding indicates that indentation deforms the membrane and underlying cytoplasm, thereby redistributing local membrane tension [31]. Under constant indentation, incremental increases in suction pressure further elevate Flipper‐TR fluorescence lifetime, indicating additional membrane stretching‐induced tension [31]. When these tension changes exceed a threshold, intracellular calcium levels increase sharply (Figure 2E) [31]. Calcium imaging signals indicate Piezo1 activation, suggesting that the channel opens when suction pressure exceeds a critical threshold [31]. This threshold varies with applied suction force, indicating that Piezo1 responsiveness is regulated by the integrated mechanical environment, including membrane tension and membrane–cytoskeleton coupling [31]. Thus, the force‐controlled nanopipette combined with Flipper‐TR provides a quantitative platform for measuring changes in cellular tension during angiogenesis and wound healing, as well as for investigating how these forces regulate vascularization through Piezo1 or other mechanosensitive pathways.

Traction forces between cells and substrates are quantified to determine the mechanical basis of cell migration and tissue contraction during wound closure [56]. Immediately after wounding, epithelial cells migrate into the wound site following the initial tissue contraction associated with the extrusion of damaged or resected cells. During the early stages of wound closure, filopodia and lamellipodia rapidly extend toward the wound edge. Approximately 15 min later, actin and myosin accumulate to form an actomyosin ring while cells remain tightly connected to neighboring cells through cell–cell adhesions [56]. TFM quantifies forces generated by migrating cells, revealing that the direction of force transmission varies with wound‐edge curvature. In convex regions, protrusive activity predominates, and traction is directed away from the wound, whereas recessed regions exhibit minimal protrusion and traction concentrated toward the wound edge, where actin accumulates [56]. As wound closure progresses, the leading edge becomes less distinct, wound‐directed traction spreads, and radial traction components increase with edge curvature [56]. Substrate stiffness also influences traction and wound closure. Even on soft 3 kPa substrates, actomyosin assembly, protrusion extension, and wound‐closure rates remain comparable to those observed on stiffer substrates, suggesting that wound‐healing responses are preserved across diverse mechanical environments through adaptive regulation of cell–substrate interactions [56].

### 2.2 Cell Junction Mechanics

#### 2.2.1 Role of Force in Cell Junction Mechanics

In collective cell migration, individual cells move autonomously while coordinating their behavior through signals transmitted via cell–cell adhesions [57]. This intercellular coordination is primarily mediated by AJs and desmosomes (Figure 1). Classical cadherins bind neighboring cells through their extracellular domains and connect to the actin cytoskeleton through plaque proteins, thereby providing mechanical stability and facilitating signal transmission [58]. Cadherin‐based cell junctions mechanically couple neighboring cells and regulate transcription, cell polarity, cytoskeletal organization, and mechanotransduction [59]. E‐cadherin, a transmembrane receptor, forms Ca^2+^‐dependent homophilic interactions with neighboring cells through its extracellular domain [60]. Its cytoplasmic domain binds α‐catenin, β‐catenin, and p120‐catenin, linking the complex to the actin cytoskeleton and providing a strong platform for signal transmission [61]. The cadherin–catenin complex functions as a mechanically coupled interface that transmits actomyosin‐generated tension [62]. At this interface, myosin II‐generated tension exposes the vinculin‐binding site on α‐catenin, thereby recruiting vinculin to stabilize cell–cell junctions [63]. These force‐dependent interactions regulate cadherin cluster size, adhesion maturation, and intercellular cohesion, maintaining epithelial integrity and coordinated cell migration [6]. In addition to cadherins, nectin, an immunoglobulin (Ig)‐like cell adhesion molecule, mediates homophilic and heterophilic interactions between neighboring cells [64]. Intracellularly, it links to the actin cytoskeleton through afadin, supporting cellular architecture and cooperating with cadherin‐based AJs to stabilize early cell–cell adhesion. This adhesion depends on interactions among nectin, cadherins, and multiple cell‐surface proteins, while adjacent cells are connected via cytoplasmic tails linking to the actin cytoskeleton through adaptor proteins such as afadin and band proteins [65]. Afadin contains an F‐actin–binding domain that links nectin to the actin cytoskeleton, distributes actomyosin‐generated forces, and regulates intercellular signaling [66]. Nectin–afadin‐mediated adhesion is essential for epithelial polarity, zygotic organization, neurogenesis, spermatogenesis, and brain development [65]. Desmosomes are transmembrane junctional complexes that provide robust intercellular adhesion, maintain tissue architecture and integrity, and resist mechanical stress [58]. Desmosomal cadherins, including desmogleins (DSGs), mediate homophilic or heterophilic interactions through their extracellular domains [67] and connect intracellularly to plakoglobin, plakophilin (PKP), and desmoplakin (DSP), thereby linking to the intermediate filament network [68]. This coordination ensures mechanical resilience, particularly in highly stable tissues, such as cardiac muscle and skin [69]. Therefore, desmosomes serve as core structural components that maintain cell adhesion, tissue architecture, and mechanical stress resistance.

At AJs, the cadherin–catenin complex initiates intercellular adhesion and couples with actomyosin contractility to promote cadherin clustering and junction maturation [70]. During this process, α‐catenin connects to E‐cadherin through β‐catenin and unfolds in response to cytoskeletal tension, exposing its central middle (M) domain and enhancing interactions with vinculin and other actin‐binding proteins [70]. Acting as a mechanosensor, α‐catenin unfolds under low tension (∼5 pN), releasing vinculin autoinhibition in response to mechanical and biochemical cues and forming a stable β‐catenin–α‐catenin–vinculin complex that transmits force to the actin cytoskeleton [64]. Clustering of E‐cadherin ectodomains alone is insufficient to stabilize cell–cell adhesion, whereas direct interactions between α‐catenin and actin improve cadherin cluster stability and junction maturation in coordination with actomyosin contractility [71]. This integration of cadherin–catenin complexes, actin cytoskeletons, and cadherin clusters locally suppresses membrane protrusions, generates junctional tension, and stabilizes AJs, thereby promoting intercellular mechanical continuity, coordinated cell migration, and tissue morphogenesis [72]. Epithelial cells are arranged side by side through adherens and tight junctions, forming a continuous monolayer within the apical junctional complex [73]. Located at the apical surface of polarized epithelial cells, this complex regulates cell polarity, adhesion, and permeability [74]. AJs directly connect to actin filaments through the cadherin–catenin complex, forming an actomyosin contractile belt that stabilizes the junction and adjacent tight junctions. Mechanical tension generated at AJs promotes actin cytoskeletal remodeling, thereby strengthening and reorganizing junction clusters. Tight junction proteins, including zonula occludens‐1 (ZO‐1), ZO‐2, junctional adhesion molecule‐A, cingulin, claudins, and occludin, link to the apical actin cytoskeleton and establish barrier function and regulate paracellular transport through mechanical coupling with AJs [74]. Consequently, the actin‐based contractile ring and adherens–tight junction connections maintain epithelial barrier integrity, cell polarity, and mechanical stability [75].

Cell–cell adhesion complexes are dynamic, mechanosensitive structures that respond to and transmit mechanical forces across cells and tissues. External mechanical cues, including extracellular substrate deformation, fluid‐induced shear stress, and tissue elongation, act on cells and induce conformational changes in junctional proteins such as cadherins and catenins. These proteins connect to the cytoskeleton, reinforcing junction stability and promoting cytoskeletal reorganization. Under tension, α‐catenin unfolds to expose a vinculin‐binding site, enabling vinculin recruitment and actin coupling that strengthen AJs and promote cytoskeleton reorganization [70]. This process exemplifies outside‐in signaling, whereby external cues are transmitted into the cell. Intercellular adhesion is maintained by AJs, which connect to the intracellular actomyosin network [76]. These structural connections provide the basis for transmitting internally generated forces to the cell–cell junctions. This force transmission creates a feedback loop that enhances intracellular cadherin binding and clustering, thereby increasing junctional stability [77]. These events represent a core mechanism of inside‐out signaling. Elevated RhoA activity strengthens actomyosin interactions, promotes stress fiber formation and AJ clustering, and increases tension transmission to junctions. Accordingly, AJs sense and transmit intracellular forces while forming a feedback loop that reinforces actomyosin‐generated tension [77]. Consequently, junction stability is enhanced, myosin‐mediated forces are generated, and the mechanical properties of the cell sheet are regulated. These inside‐out signaling feedback mechanisms are closely associated with physiological processes, including junction remodeling, reduced protrusions, cell attachment maintenance, tissue morphogenesis, and wound healing.

#### 2.2.2 Physiological and Pathological Applications for Analyzing Junction Mechanics

Cells regulate biological processes—including development, homeostasis, migration, and tissue formation—via sensing and responding to mechanical forces from the extracellular environment and intracellular compartments. Even pN forces can induce key structural and functional changes, including modulation of Notch signaling, changes in actin crosslinker binding, and stabilization of cell adhesion complexes [78]. This process commonly occurs in vivo at force levels comparable to those generated via cytoskeletal motor proteins at the single‐molecule scale [78]. FRET is a fluorescence‐based technique used to detect Nanometer‐scale distance changes induced via mechanical tension. FRET occurs when energy from an excited donor fluorophore is nonradiatively transferred to a nearby acceptor fluorophore, with transfer efficiency determined based on intermolecular distance, relative orientation, and spectral overlap [32]. FRET efficiency decreases sharply with increasing intermolecular distance and can be quantified using fluorescence intensity ratios or donor fluorescence lifetime measurements [32]. Based on this principle, tension sensors are generated via inserting a force‐responsive protein or peptide between donor and acceptor fluorophores, enabling conformational extension or structural rearrangement in response to mechanical tension. These sensors can be fused to intracellular or cell‐surface proteins to monitor local molecular tension in real time via reductions in FRET efficiency at specific sites. Fluorescent protein‐based donor–acceptor pairs can be genetically encoded to directly report mechanical force changes during processes such as cell signaling and intercellular adhesion [32].

Loss or downregulation of E‐cadherin disrupts epithelial structural integrity and promotes mechanical and biochemical dedifferentiation. Reduced E‐cadherin expression enhances actomyosin contractility while weakening membrane–cytoskeletal coupling, leading to asymmetric distributions of intracellular and cortical tension [79]. Simultaneously, the PI3K/AKT pathway becomes aberrantly activated, promoting anchorage‐independent growth and resistance to anoikis, enabling invasive lobular carcinoma cells to evade ECM attachment–dependent death signaling and survive during dissemination through the bloodstream and other body fluids [79]. These findings suggest that E‐cadherin loss contributes to mechanical instability and altered metastatic behavior in cancer cells.

**FIGURE 3:**
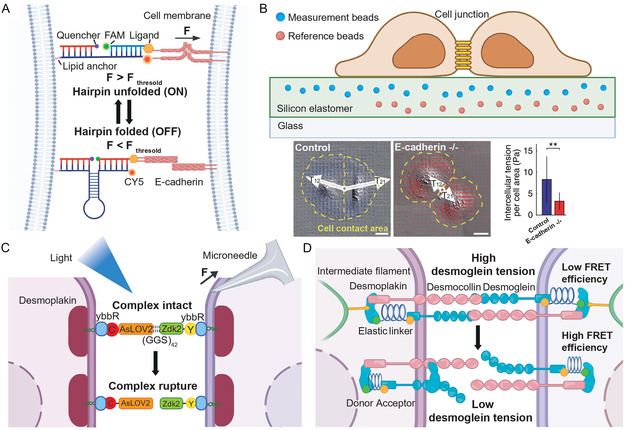
Quantification of mechanical forces transmitted across cell–cell adhesions. (A) Conceptual schematic of lipid‐modified DNA molecular tension probes. A DNA hairpin with a defined force threshold is flanked by a fluorophore (FAM) and a quencher (Eclipse) at opposite ends. Lipid modification anchors the probe in the cell membrane, while a protein G linker enables selective binding to E‐cadherin. Tensile force generated at cell–cell adhesions unfolds the hairpin, separating FAM and quencher and restoring fluorescence. A Cy5 reference signal enables normalization independent of tension‐dependent fluorescence changes. Adapted with permission [80] 2023, *Frontiers in Cell and Developmental Biology*. (B) Conceptual schematic of TFM. Two layers of fluorescent beads are embedded within an elastic substrate. One layer serves as a fiduciary reference at the glass–elastomer interface, while the second layer tracks cell‐induced deformation. Lower image: a traction stress map. Bead displacements measured before and after cell detachment are used to calculate traction stresses using the Fourier transform TFM. Blue and red arrows indicate local in‐plane traction stress vectors (σz) exerted by cells on the substrate, reflecting actomyosin‐driven cell–substrate forces. White arrows denote the resultant intercellular tension vectors derived from vectorial summation of traction stresses within the dashed boundary of adjacent cells, representing net force transmission across the cell–cell junction. The graph shows intercellular tension per unit area, calculated by normalizing total tension to cell area. Adapted with permission [6] 2017, *Nature Communications*. (C) Conceptual schematic of optomechanical FRET‐based molecular control. The light‐sensitive AsLOV2–Zdk2 system is inserted into desmoplakin, splitting it into two mechanically linked fragments (AsLOV2, Zdk2) connected by a short, flexible GGS linker. In the dark, the AsLOV2–Zdk2 interaction remains stable. Illumination with 458 nm light induces rapid and reversible dissociation of the complex, selectively weakening the mechanical linkage within the desmoplakin. Under this optically weakened state, externally applied mechanical stress via microneedle manipulation increases bond rupture frequency at desmoplakin‐mediated junctions. This system demonstrates that junctional stability depends on both optically controlled molecular interactions and externally applied mechanical forces. Adapted with permission [81] 2023, *Science Advances*. (D) Conceptual schematic of a FRET‐based Dsg3 tension sensor. The sensor comprises an mTFP (donor fluorophore) and Venus (acceptor fluorophore) pair inserted into the Dsg3 cytoplasmic tail and connected using an elastic linker (PM). Mechanical tension stretches the linker, increasing the donor–acceptor distance and decreasing FRET efficiency. Adapted with permission [82] 2025, *Iscience*. AsLOV2, *Avena sativa* light‐oxygen‐voltage‐sensing domain; Cy5, cyanine 5 reference dye; Dsg3, desmoglein 3; E‐cadherin, epithelial cadherin; FAM, fluorophore amidite; FRET, Förster resonance energy transfer; GGS, glycine–glycine–serine linker; mTFP, monomeric teal fluorescent protein; PM, proline–methionine linker; TFM, traction force microscopy; Venus, yellow fluorescent protein variant.

An unsaturated, anchor lipid‐modified DNA molecular tension probe was used to visualize and validate E‐cadherin‐mediated intercellular tension [80]. This modular DNA probe consists of a lipid‐anchored strand, a ligand strand that targets specific cell adhesion molecules, and a force‐responsive DNA hairpin (Figure 3A) [80]. Cholesterol on the anchor strand spontaneously inserts into the membrane lipid bilayer, stably anchoring the probe without genetic modification or complex surface functionalization [80]. The ligand strand binds cell–cell adhesion molecules, including E‐cadherin, directing the probe to specific intercellular junctions. When tensile forces across cell–cell adhesions exceed the hairpin unfolding threshold, the hairpin unfolds, separating the fluorophore from the quencher and increasing fluorescence intensity (Figure 3A). Furthermore, co‐introducing a force‐insensitive reference dye (Cy5) enables ratiometric analysis to normalize force‐dependent signal changes and minimize effects of probe density or local concentration variability [80]. The 6‐carboxyfluorescein (FAM)/Cy5 fluorescence ratio quantitatively reports force‐induced DNA hairpin unfolding. DNA hairpin‐based tension probes (*F*_threshold_ = 4.4 pN; 22% GC content) are used to measure intercellular forces in MCF‐7 tumor spheroids [80]. Compared with the control probe lacking a quencher (NQ probe), the Q probe exhibits a lower FAM/Cy5 ratio, confirming efficient fluorescence quenching in the unstressed state [80]. Conversely, the E‐cadherin‐bound probe shows a significantly higher FAM/Cy5 ratio than that of the Q probe, indicating the presence of E‐cadherin‐mediated tensile forces in MCF‐7 spheroids [80]. A key advantage of this technology is the ability to tune molecular tension probe force thresholds via DNA sequence–dependent control of hairpin stability. This approach enables selective detection of mechanical forces associated with specific ligand–receptor interactions.

MTs were used to analyze E‐cadherin‐mediated adhesion and its effects on cellular adhesive force. In WT epidermoid carcinoma (A431) cells, E‐cadherin‐coated beads exhibit strong and stable adhesion [83]. Approximately 70% of the beads remain attached after application of a 0.5 nN tensile force pulse [83]. Contact with CAFs induces strong adhesion and mechanical reinforcement at E‐cadherin/N‐cadherin junctions. In high‐frequency MT pulse experiments, WT A431 cells and CAFs show progressively reduced bead displacement and strengthened adhesion via E‐cadherin/N‐cadherin junctions [83]. Conversely, E‐cadherin‐deficient cells show no reduction in bead displacement, confirming that adhesion strengthening depends on E‐cadherin [83]. These findings quantitatively demonstrate that E‐cadherin is essential for maintaining cell–cell adhesion and mechanical signaling. E‐cadherin loss weakens adhesion and disrupts force transmission during heterotypic interactions with CAFs, providing insight into how cancer cells retain epithelial traits while migrating collectively [83]. Single‐cell bead adhesion force measurements using MTs provide a quantitative approach for analyzing E‐cadherin‐dependent mechanics and may serve as a useful tool for future CAF‐cancer cell studies.

AFM was used to measure viscoelastic and stress‐relaxation properties of breast cancer (MCF‐7) cells, which were subsequently clustered using self‐organizing maps [84]. These measurements reveal that cancer cells exhibit altered mechanical phenotypes, including changes in stiffness and viscosity compared with normal epithelial cells, confirming drug‐induced alterations in cellular rigidity and viscoelastic behavior. Cells with reduced E‐cadherin expression exhibit decreased cortical stiffness and altered stress relaxation in AFM measurements, correlating with metastatic potential [84]. AFM‐based viscoelastic measurements combined with self‐organizing map analysis quantitatively characterize cancer cell mechanics and may support cancer diagnosis and metastasis assessment via adhesion‐related changes, including E‐cadherin status.

Fibrosis commonly represents a terminal stage of diverse organ diseases, including pulmonary fibrosis, congestive heart failure, cirrhosis, and end‐stage kidney disease, and is associated with progressive functional decline. Myofibroblasts are central mediators of fibrosis and differentiate from multiple progenitor cell types, including monocytes, fibroblasts, epithelial cells, and hepatocytes [85]. Myofibroblasts express contractile cytoskeletal proteins, including α‐SMA, promote ECM accumulation, increase tissue stiffness via intracellular tension, and mediate scar formation and maintenance [85]. Fibrosis is strongly regulated via biochemical signaling and mechanical cues, including substrate stiffness. Mechanical sensing is mediated via the integrin–cytoskeleton–actomyosin network and regulated via focal adhesion maturation and force‐sensitive protein interactions [85].

To quantify changes in adhesion stability, TFM was used to measure intracellular tension distribution. This approach was used to analyze how E‐cadherin deficiency affects intercellular force transmission between paired cells. The substrate contained fluorescent beads distributed in two diffuse layers [6]. One layer served as a reference between the glass and elastomer, while the second layer, located just below the gel surface, detected cell‐induced deformation (Figure 3B) [6]. After cell culture, bead positions deformed based on cell attachment were imaged before and after cell removal to quantify bead displacement. Traction stress exerted via each cell on the substrate was calculated from bead displacement data using a standard Fourier transform–based method [6]. When two cells were in contact, the vector tension on cell 1 was defined as the combined traction stress transmitted from cell 2 beneath it [6]. Similarly, the vector sum of traction stress beneath cell 2 was defined as the vector tension exerted via cell 1 on cell 2 (Figure 3B) [6]. Intercellular tension per unit cell area was calculated by dividing the measured tension by the spread area of each cell (Figure 3B) [6]. In keratinocytes, actomyosin contraction maintains uniform tension distribution at E‐cadherin‐mediated AJs, while E‐cadherin deficiency disrupts tension balance and produces localized stress concentration (Figure 3B) [6]. Additionally, the elastic modulus of the cell cortex was measured using AFM‐based force–indentation spectroscopy. E‐cadherin‐deficient cells exhibit significantly decreased cortical stiffness compared to the control cells, directly correlating with decreased intercellular tension transfer [6].

A rigid microenvironment activates mechanosensitive ion channels in the cell membrane, thereby modulating intracellular calcium signaling. Piezo1—a mechanosensitive channel that mediates Ca^2+^ influx in response to membrane deformation—is a key regulator of fibroblast activation, collagen production, and myofibroblast differentiation [86]. AFM and OT were used to quantify the mechanical responsiveness of cardiac fibroblasts cultured on substrates of varying stiffness. Compared with a stiff substrate, culturing cells on a softer polydimethylsiloxane substrate (88 kPa) significantly reduces α‐SMA expression, suppressing myofibroblast differentiation [86]. AFM force–indentation spectroscopy reveals a reduced cellular elastic modulus, confirming decreased cortical rigidity [86]. These findings suggest that insufficient substrate softening fails to generate the membrane tension required for Piezo1 activation. OT–mediated application of local forces ≤50 pN minimally induces calcium signaling, while AFM‐applied forces of 150–350 nN trigger stepwise calcium responses [86]. These findings indicate that increased substrate stiffness enhances Piezo1 expression and calcium responsiveness, while soft substrates reduce Piezo1 activity and cellular mechanosensitivity. Collectively, these findings suggest that Piezo1‐dependent signaling regulates cellular mechanosensitivity in response to ECM mechanical properties. Consequently, quantitative analyses using AFM and OT reveal that substrate stiffness directly influences cardiac fibroblast mechanosensitivity and Piezo1‐mediated calcium signaling. Notably, complementary analyses using TFM and AFM further demonstrate that E‐cadherin functions not only as an adhesion molecule but also as a mechanosensor that detects ECM rigidity and regulates intercellular tension.

AJs and tight junctions maintain epithelial tissue homeostasis, ensuring structural continuity and mechanical stability [87]. Intercellular adhesion physically connects neighboring cells and functions as a cytoskeleton‐associated mechanotransduction system. E‐cadherin connects β‐catenin to actin filaments, maintaining homeostatic tension, essential for signal transduction and regulation of cell motility [88]. Pathological stimuli, including inflammation and wound induction, destabilize intercellular adhesion proteins via phosphorylation and structural rearrangement [52]. This disruption creates cytoskeletal tension imbalance, weakens intercellular binding forces, induces asymmetric stress distribution, and reduces barrier function [52]. These mechanical changes enhance epithelial cell motility and initiate early repair processes, including re‐epithelialization [89]. Fibroblast growth factor 2 (FGF2) was used to analyze changes in keratinocyte adhesion complexes within a wound microenvironment. Immunofluorescence staining reveals significantly reduced membrane‐associated E‐cadherin signals at the wound edge, along with co‐expression of vimentin and cytokeratin [89]. These findings suggest that keratinocytes undergo partial epithelial–mesenchymal transition (EMT), promoting increased motility and migration toward the wound center [89]. In the FGF2‐treated group, β‐catenin translocates from the cell membrane to the cytoplasm and nucleus, indicating dissociation of the E‐cadherin–β‐catenin complex and activation of Wnt/β‐catenin signaling [89]. Consequently, these changes are associated with weakened intercellular adhesion and increased motility and are interpreted as a mechanical adaptation characterized by transient disassembly of intercellular junctions during wound healing to promote epithelial restoration through cell migration [89]. These changes further suggest that inflammatory responses involve biochemical signaling and mechanical disequilibrium between the cytoskeleton and adhesion complexes.

To investigate mechanical forces regulation during cell–cell adhesion, MTs can be used to quantitatively assess local mechanical responses of adhesion proteins. This technique enables real‐time analysis of cellular mechanical behavior via coating magnetic beads with specific adhesion proteins, attaching them to cell‐surface receptors, applying localized tensile forces, and tracking bead displacement trajectories [90]. Following attachment of E‐cadherin‐coated magnetic beads to the cell surface, an oscillatory tensile force was applied. Under force application, bead displacement amplitude gradually decreases, indicating that cells strengthen structural connections through adhesion‐mediated mechanoadaptation in response to external forces [90]. Progressive increases in tensile force reinforce E‐cadherin‐mediated adhesion in a force‐dependent manner [88]. Additionally, reducing E‐cadherin density on bead surfaces decreases adhesion stiffness [90]. Vinculin localization at cell–bead contact sites was also assessed. Tensile stimulation of E‐cadherin promotes marked vinculin accumulation at these sites, indicating force‐dependent adhesion reinforcement [90]. Additionally, vinculin knockdown significantly weakens cellular reinforcement responses [90]. Thus, E‐cadherin mediates active structural reinforcement in response to tensile force. The mechanical properties of cadherins are crucial for coordinating intercellular tension, cell motility, and epithelial wound closure and provide insight into the interplay between mechanical heterogeneity and adhesion complex dynamics.

Cardiomyopathy is a primary cardiac disease associated with abnormal adhesion between cardiomyocytes. Desmosomes and AJs are key structures that maintain mechanical and electrical coupling between cardiomyocytes during disease progression. In the heart, desmosomes preserve mechanical continuity between adjacent cells and transmit contraction‐generated tension. Approximately 50% of patients with arrhythmogenic cardiomyopathy (ACM) carry mutations in desmosome‐related genes—including PKP2, DSP, DSG, and desmocollin (DSC)—with PKP2 mutations being the most prevalent [13]. Mutations that reduce desmosomal protein stability and binding weaken intercellular mechanical adhesion, leading to cell loss, impaired electrical coupling, and increased myocardial instability [13]. Loss of desmosomal proteins also directly disrupts AJ protein complexes. Major AJ proteins—β‐catenin, N‐cadherin, and α‐catenin—are consistently reduced in patients with ACM and in PKP2‐mutant mouse hearts, while immunostaining reveals disrupted protein alignment and widespread dispersion [13]. Additionally, AJ protein expression closely correlates with PKP2 levels, suggesting functional interdependence between the two complexes [13]. Therefore, restoring desmosome–AJ stability and intercellular adhesion via proteolysis regulation remains crucial for cardiomyopathy management.

DSP is the key protein that connects cadherins and intermediate filaments to desmosomes. An optogenetic dimerization‐based system was used to evaluate the role of DSP‐mediated mechanical coupling in intracellular force transmission and intercellular adhesion integrity [81]. DSP was divided into two fragments and expressed as mCherry‐AsLOV2 and Zdk2‐YPet‐DspII and then introduced into DSP‐deficient mouse epithelial keratinocytes (Figure 3C) [81]. The two DSP fragments were engineered to reversibly associate or dissociate in response to light [81]. In differentiated cells, they efficiently localize to desmosomes, and electron microscopy confirms intact intermediate filament connections [81]. Live‐cell imaging further shows that the DSP fragments are dispersed before differentiation but localize and co‐localize at intercellular junctions after differentiation [81]. To evaluate DSP function under mechanical stress, cells were mechanically stretched using a fine needle at 1 μm/s to apply force to intercellular junctions. This exposes cells to molecular‐scale forces comparable to those generated by motor proteins under natural conditions. Simultaneous light stimulation rapidly disrupts DSP interactions and triggers the rapid collapse of intercellular junctions [81]. Although DSP is not required to maintain cell–cell junctions under homeostatic conditions, it is essential for preserving junctional mechanical integrity under applied force. Additionally, light‐induced reversible molecular dissociation directly shows that DSP‐mediated mechanical connections are critical for maintaining intercellular cohesion under external stress. Molecular‐scale force regulation and molecular dissociation highlight the critical role of desmosome–keratin connections in tissue integrity and in mechanically driven disease injury.

DSG2, the sole desmoglein expressed in cardiac desmosomes, is essential for maintaining stable adhesion between cardiomyocytes. DSG2 gene variants are associated with arrhythmogenic right ventricular cardiomyopathy (ARVC), a disorder characterized by cardiomyocyte loss and fibrofatty replacement of the ventricular myocardium [91]. The effects of WT DSG2 and two ARVC‐associated mutations on heterophilic binding and intercellular adhesion were assessed at single‐molecule and cellular levels. Single‐molecule analysis was conducted using AFM‐based single‐molecule force spectroscopy (SMFS). This technique directly quantifies bond strength, kinetic parameters, and thermodynamic properties between individual molecules. The DSG2 extracellular domain (EC1–4) was immobilized on the AFM cantilever tip and substrate. PEG linkers prevent direct molecular contact and maintain appropriate spacing between the interacting molecules. A specific bond forms between DSG2 molecules as the AFM probe approaches the substrate [91]. Retraction of the probe generates a force that dissociates the bond between the two DSG2 molecules [91].

Over 10,000 force–distance cycles were recorded across multiple tensile loading rates, and separation distances generated from forces applied to the DNA unzipping (DU) probe were used to construct force–distance curves [91]. This approach enables quantitative comparison of single‐molecule binding interactions between WT DSG2 and ARVC‐associated DSG2 mutants. Force–failure behavior and molecular elasticity were analyzed using the Evans–Ritchie model to estimate bond dynamics and thermodynamic parameters, including the dissociation rate constant (k_off) and reaction length (x_β) [91].

AFM‐SMFS analysis shows that WT DSG2 exhibits an average bond lifetime of ∼0.33 s. The p.D105E (p.D154E) and p.V343I (p.V392I) mutants increase bond lifetime through reduced N‐terminal strand mobility due to disrupted Ca^2+^ coordination between EC1 and EC2. This disruption delays strand exchange and reduces the rate of molecular association [91]. In contrast, the other mutant shows no significant difference relative to WT, likely due to preserved homophilic binding interactions [91]. These findings suggest that the mutations do not significantly alter bond free energy but affect bond dynamics—such as bond lifetime and reaction length—thereby influencing adhesion regulation. AFM‐SMFS analysis further shows that these mutations prolong binding lifetime and modify binding kinetics, resulting in enhanced cellular adhesion. This process is associated with highly conserved desmosomal protein sequences and spatial constraints within the adhesion complex. The study further demonstrates that mutant DSG2 cadherin influences adhesion regulation and cellular network stability.

AJs and desmosomes primarily mediate adhesion between epidermal keratinocytes, with desmosomes providing the majority of mechanical stability [58]. Desmosomes contain cadherin family proteins, including DSGs and DSCs, which connect intermediate filaments to intracellular keratin networks through plaque proteins [92]. In the epidermis, DSG1 expression is low in the basal layer and progressively increases across the suprabasal layers toward the stratum corneum [58].

In patients with chickenpox, autoantibodies target DSG3 and other DSGs, disrupting normal intercellular adhesion. This disruption weakens the adhesion between epidermal keratinocytes, leading to cell dissociation and blister formation [93]. The functional redundancy and stratified expression patterns of DSG proteins are essential for epidermal homeostasis. Studies examining DSG3‐mediated junctional dysfunction in the presence of pemphigus vulgaris (PV) antibodies show that desmosomal cadherins contribute to intercellular adhesion, mechanical resilience, and signaling pathways involved in stem cell maintenance and differentiation [58]. Thus, DSG1/DSG3 autoantibodies disrupt desmosomal integrity, leading to pathological alterations in intraepidermal junctional architecture and signaling networks contributing to skin blister formation.

A custom FRET‐based sensor was engineered to directly measure tension across DSG3 by inserting a molecular tension sensor module (TSMod) into the protein. The TSMod consists of a donor fluorophore, monomeric teal fluorescent protein, and an acceptor fluorescent protein, Venus, connected via an elastic peptide linker extending or relaxing in response to externally applied force (Figure 3D) [82]. This sensor module was inserted into the cytoplasmic domain of DSG3, enabling fluorescence signals to directly reflect mechanical tension across the protein. Tensile force stretches DSG3, increasing the distance between the donor and acceptor fluorophores within the TSMod and reducing FRET efficiency [82]. Conversely, reduced tension allows DSG3 to relax, decreasing the distance between the fluorophores and increasing FRET efficiency (Figure 3D). Therefore, an increased FRET ratio indicates reduced mechanical tension across DSG3, suggesting diminished force‐bearing capacity at cell–cell junctions [82]. This tension arises from intrinsic mechanical forces generated at cell–cell junctions and sustained by cytoskeletal and desmosomal interactions rather than external mechanical stress.

The tailless sensor retains the extracellular and transmembrane domains of DSG3 while uncoupling the protein from the cytoskeleton [82]. This design prevents indirect tension transfer associated with cellular contractility or cytoskeletal rearrangement [82]. PV‐derived antibodies significantly increase the FRET ratio in the full‐length DSG3 FRET sensor, while the tailless sensor shows no significant change [82]. This finding strongly suggests that the PV antibody‐induced increase in FRET reflects a loss of intrinsic tension across DSG3 rather than structural changes in the sensor or nonspecific experimental effects.

Additionally, the DSG3 FRET sensor was combined with TFM to evaluate mechanical tension at the molecular and whole‐cell levels. These findings indicate that PV antibody treatment reduces DSG3 molecular tension and is associated with altered cell–cell adhesion stability and changes in force distribution between cells and the extracellular substrate [82]. These findings suggest that desmosomal disruption in PV pathophysiology arises from loss of molecular tension at the cell–cell junctions, reflecting impaired mechanotransduction rather than simple depletion of adhesion molecules [82]. The DSG3‐specific FRET tension sensor enables real‐time measurement of intrinsic tension across cell–cell junction proteins, offering key insights into the mechanical homeostasis of desmosome‐mediated adhesion and its molecular‐scale disruption. Future application of this approach to diverse cell–cell adhesion proteins may advance understanding of tissue stability, disease pathogenesis, and the molecular mechanisms underlying mechanotransduction.

## 3 Force Transmission to the Nucleus

### 3.1 Mechanical Properties of the Nuclear Membrane

Among intracellular organelles, the nucleus is the largest and mechanically stiffest structure and is directly exposed to mechanical forces from intracellular and extracellular environments [94]. A double‐membrane lipid bilayer—termed the NE—surrounds the nucleus and comprises the INM and ONM [95]. The ONM is continuous with the endoplasmic reticulum (ER), participates in protein synthesis, and connects to the cytoskeleton to facilitate mechanical force transmission [96]. In contrast, the INM contains proteins such as emerin and directly interacts with lamins and chromatin to maintain nuclear structural integrity and regulate gene expression [96].

The INM and ONM are connected through the nuclear pore complex (NPC) [97]. The NPC is a large protein assembly ∼100 nm in diameter with a cylindrical, octagonally symmetric structure composed of three distinct rings: cytoplasmic, inner, and nuclear rings [98]. NPCs regulate molecular transport between the nucleus and cytoplasm and contribute to nuclear mechanosensing through force‐induced changes in permeability [95]. Increased mechanical tension enhances NPC permeability, facilitating nuclear import of mechanosensitive transcriptional regulators such as YAP and myogenic differentiation factors [96].

The NE maintains close connections with the cytoskeleton and serves as a central platform for transmitting mechanical forces [99]. The LINC complex primarily mediates this connection. The LINC complex comprises interactions between Sad1 and UNC‐84 (SUN)‐domain proteins in the INM and nesprins in the ONM, with nesprins connecting to actin filaments, microtubules, and intermediate filaments in the cytoplasm [99]. Consequently, mechanical forces originating from the ECM are transmitted via the cytoskeleton to the nuclear lamina and chromatin.

Lamins—particularly lamin A/C—provide structural support to the NE, connect the nucleus to the cytoskeleton, and regulate transcription, chromatin organization, and DNA repair [100]. Emerin—a key protein localized to the INM and ONM—interacts with lamins to maintain nuclear stability and support force transmission and cellular polarity [99]. Emerin deficiency alters migratory behavior, including increased migration persistence, reduced local adhesion, and impaired force transmission to the extracellular substrate [99]. Emerin functions beyond a purely structural role and contributes to regulating the directionality and efficiency of cellular force transmission. Collectively, these findings suggest that the NE functions as a physical barrier and a critical mechanical interface connecting the ECM, cytoskeleton, nucleus, and chromatin. This positioning enables the nucleus to act as a mechanosensor that detects the cellular mechanical environment and regulates gene expression and cell fate [96].

The NE comprises a double‐membrane lipid bilayer supported by the underlying nuclear lamina, serving as a structural boundary and scaffold for transmitting intracellular and extracellular mechanical forces [101]. The nuclear lamina is a 15–60 nm protein network composed primarily of lamins [14]. Lamins are type *V* intermediate filament proteins containing a central α‐helical coiled–coil domain and intrinsically disordered N‐ and C‐terminal regions, including an Ig‐like fold within the C‐terminus [102]. These structural features promote lateral interactions between lamin molecules, forming flexible filaments ∼400 nm in length [14].

The nuclear lamina comprises lamin A/C and B‐type lamins (lamin B1 and lamin B2), encoded by the LMNA and LMNB1/LMNB2 genes, respectively [103]. A‐type lamins are predominantly expressed in differentiated cells and mechanically stiff tissues, where they function as mechanical buffers against external forces [104]. In contrast, B‐type lamins are essential for organ development and are expressed in nearly all cell types [104]. These functional differences indicate that lamins differentially regulate nuclear mechanical properties in a cell type‐ and developmental stage‐dependent manner, extending beyond their role as structural scaffolds.

The nuclear lamina connects to the cytoskeleton via the LINC complex [14]. Actin‐ and myosin II‐generated contractile forces propagate through the F‐actin network and transmit to the NE via the LINC complex [104]. Lamin proteins absorb mechanical tension and distribute forces within the nucleus, thereby preserving nuclear integrity [103]. Collectively, these findings suggest that the nuclear lamina contributes to the transmission and spatial distribution of mechanical signals between the cytoplasm and chromatin.

Chromatin interacts with lamins to form LADs, which are chromatin regions anchored to the NE [105]. LADs comprise primarily heterochromatin and maintain transcriptional repression while defining functional genomic domains [105]. LAD formation and reorganization are crucial for cellular development and differentiation, and external mechanical forces transmitted via lamins induce chromosomal rearrangements that regulate gene expression [103]. Lamin mutations or dysregulated lamin expression compromise nuclear mechanical stability. For example, lamin A mutations are associated with disorders such as Hutchinson–Gilford progeria syndrome and Emery–Dreifuss muscular dystrophy, altering nuclear stiffness through abnormal increases or decreases [9].

Conversely, elevated lamin A expression enhances nuclear viscoelasticity and stiffness, contributing to tissue‐level mechanical adaptability [9]. Collectively, the nuclear lamina functions as a physical interface linking external mechanical forces to intracellular gene expression programs rather than serving solely as a structural scaffold [14]. Interactions between lamins and LADs maintain nuclear stability and integrate mechanical signals into chromatin reorganization and transcriptional regulation during cellular differentiation and development [14].

NE wrinkling is associated with biological processes such as nuclear positioning. The NE also functions as a mechanosensor that regulates chromatin dynamics and transcription factor transport through NPCs [106]. NE wrinkling may result from nuclear growth and reduced lamin C expression and serves as a tension‐buffering mechanism [106]. External mechanical forces first promote NE wrinkling, followed by in‐plane deformation that induces nuclear rupture [106]. This two‐step process also occurs during cell migration, where stretch‐mediated responses emerge after partial nuclear flattening [107]. Microtubule‐generated forces at the nuclear surface induce nuclear lamina wrinkling [108].

The nuclear droplet model assumes that the mammalian nucleus has a larger surface area than a sphere of equal volume [108]. Therefore, a spherical nucleus inherently contains wrinkles that spread as the nucleus deviates from a rounded morphology. Cellular structural changes ultimately regulate nuclear wrinkling [109]. Nuclear stability depends on the mechanical resistance of the nuclear lamina and the compressive resistance of nuclear contents [109]. Two‐dimensional (2D) hydrogel experiments demonstrate that most nuclear wrinkles are attributed to the NE rather than direct cytoskeletal collisions [107].

Wrinkled nuclei typically exhibit irregular, undulating morphologies similar to those observed during early stages of cell attachment and detachment. NE wrinkle dynamics are highly sensitive to changes in cellular contractility [107]. Cells cultured on stiff substrates generally exhibit smooth, unwrinkled nuclei, while moderate nuclear wrinkling occurs on 10 kPa 2D hydrogels. Pharmacological studies show that increased cellular contractility eliminates nuclear wrinkles and strains, while wrinkle formation occurs independently of transcription and translation [110]. These findings suggest that NE wrinkles act as an immediate mechanical response that dissipates deformation energy [107].

NE wrinkling is tightly associated with mechanotransductive signaling. Wrinkle‐free nuclei under mechanical strain exhibit enhanced YAP/TAZ nuclear localization, whereas wrinkled nuclei show lower nuclear‐to‐cytoplasmic YAP/TAZ ratios and reduced transcriptional activity [107]. Collectively, NE wrinkles represent dynamic, mechanically responsive nuclear membrane structures associated with rapid tension buffering, chromatin reorganization, transcriptional regulation, and cell fate determination [102]. These properties are central to understanding how cytoskeletal forces integrate with nuclear architecture and genomic function and are relevant to diseases involving impaired development, differentiation, and nuclear dynamics.

Cytoskeletal mechanical stress is transmitted to the nucleus via the LINC complex embedded in the NE [111]. The LINC complex comprises SUN1/2 protein spanning the INM and nesprin proteins localized to the ONM, which interact directly within the NE lumen [112]. The cytoplasmic nesprin domain connects to multiple cytoskeletal components, including actin filaments and microtubule‐associated motor proteins, while the Klarsicht/ANC‐1/Syne homology domain interacts with SUN proteins [113]. Conversely, SUN proteins mechanically connect the cytoskeleton to the nucleoskeleton through direct interactions with lamins and chromatin [113]. LINC complexes contribute to diverse biological processes, including cell migration, nuclear positioning, nucleo‐cytoskeletal linkage maintenance, DNA damage repair, and meiotic chromosome movement [113].

Myosin‐generated contractile forces are essential for cellular sensing of substrate stiffness [111]. Actomyosin‐generated tension is transmitted to the nuclear surface via the LINC complex, and nonmuscle myosin II inhibition alters gene expression patterns similar to those observed after LINC complex disruption [111]. These findings suggest that the LINC complex regulates mechanical coupling and mechanotransduction pathways, including transcriptional control [111]. Genes commonly affected by myosin inhibition and LINC complex disruption define a mechanosensitive gene set directly dependent on actomyosin‐generated nuclear tension [114, 115].

Within the nucleus, the LINC complex connects to the nuclear lamina along the inner surface of the NE. The nuclear lamina comprises a ∼15 nm‐thick protein meshwork composed of A‐ and B‐type lamin filaments that determines nuclear mechanical strength [14]. Most chromatin is associated with the nuclear lamina, and LADs closely regulate gene expression. Lamin A/C is distributed throughout the nuclear lamina and interacts with chromatin to regulate chromatin accessibility and spatial organization. External mechanical stress alters local chromatin organization and transcription in a lamin A/C‐dependent manner, indicating that the NE–lamina–chromatin system functions as a continuous axis for mechanical signal transmission [14]. Therefore, the integrated network of LINC complexes and the nuclear lamina serves as a central pathway connecting the cytoskeleton and genome [14].

### 3.2 Intracellular Nuclear Mechanics

Within the nuclear mechanical environment, chromatin functions as an active mechanosensitive system that responds to intracellular and extracellular mechanical stimuli rather than a passive structural component. Chromatin compaction, histone modifications, structural remodeling, and spatial organization collectively determine nuclear viscoelastic properties and regulate nuclear deformation, DNA damage responses, and gene expression under mechanical stress [116]. Chromatin, organized into heterochromatin and euchromatin, regulates nuclear viscoelastic properties through its compaction state [105]. Heterochromatin forms mechanically stable nuclear domains that buffer external stress and limit DNA damage [117]. Conversely, euchromatin is less compact, promoting mechanical compliance, stress dissipation, and rapid transcriptional activation [117].

External mechanical forces are transmitted to the NE via the cytoskeleton and LINC complex, while chromatin modulates deformation of specific genomic regions through compaction‐dependent regulation of force propagation [8]. For example, heterochromatin‐rich regions near the nuclear periphery show less deformation under mechanical stimulation, while centrally localized euchromatin undergoes greater deformation and chromatin remodeling. These observations suggest that localized nuclear deformation serves as a key mechanism connecting mechanical signaling to gene expression regulation.

Chromatin‐mediated nuclear mechanical protection involves two primary mechanisms. The first is a rapid, Ca^2+^‐dependent response triggered by ER Ca^2+^ release. ER Ca^2+^ signaling decreases lamina‐associated heterochromatin, increases chromatin mobility, and reduces NE tension [117]. These responses alleviate DNA mechanical stress induced by sudden external forces, while chromatin fluidization dissipates mechanical energy and prevents DNA twisting or breakage.

The second mechanism is a slower, less sensitive response that limits nuclear deformation by readjusting cellular and nuclear long axes, adhesion sites, and F‐actin networks. This process depends on intercellular contacts and responds to sustained, tissue‐level mechanical forces. Gradual structural reorganization enables nuclei and cells to maintain stability under sustained mechanical stress [117]. These two protective mechanisms regulate nuclear dynamics through changes in chromatin state and provide rapid buffering against acute mechanical stress [117].

Mechanical force–induced deformation is associated with chromatin reorganization and epigenetic remodeling [117]. Under mechanical stress, repressive histone marks such as histone H3 lysine 9 trimethylation (H3K9me3) and H3K27me3 are redistributed or reduced, increasing euchromatin accessibility and enhancing transcription [116]. Conversely, cells cultured on highly viscoelastic substrates exhibit increased chromatin compaction, repressive histone mark retention, and enhanced nuclear stability. Viscoelastic chromatin interactions dynamically regulate gene expression in response to extracellular environmental cues.

Chromatin condensation directly influences nuclear morphology. Loosely packed euchromatin promotes NE wrinkling and reduces intranuclear pressure, facilitating deformation [116]. Conversely, highly condensed heterochromatin reinforces nuclear structural integrity and increases resistance to external mechanical forces. In this context, lamin A/C provides limited structural support, while nuclear stability depends largely on chromatin organization and compaction [8]. Chromatin functions as an active mechanosensor and mechanical buffer, protecting the nucleus and DNA from mechanical stress while integrating nuclear viscoelasticity, epigenetic states, and morphological stability.

The strength and organization of lamin–chromatin interactions determine NE mechanical properties and intranuclear stress distribution [118]. Lamin A is present in the NE and nucleoplasm, binding chromatin directly or via histone complexes to crosslink chromosomes [119]. These interactions generate subdiffusive chromatin dynamics, leading the nucleus to exhibit viscoelastic responses to external mechanical forces [119]. Conversely, lamin A deficiency rapidly shifts chromatin dynamics from constrained, slow diffusion to near‐normal diffusive behavior [119].

Weakened lamin–chromatin interactions increase NE susceptibility to external deformation driven by heterogeneous internal stress [105]. Loss or reduction of lamin A disrupts peripheral heterochromatin anchoring and weakens NE–chromatin coupling, leading to NE undulations or localized rupture [118]. These structural changes reduce overall nuclear viscoelasticity, generate heterogeneous intranuclear stress distributions, and impair force transmission from the cell exterior to the nucleus [118].

FRET‐based lamin tension sensor studies indicate that lamin A/C tension varies across cellular conditions, including the cell cycle, EMT, and chromatin reorganization [14]. These findings suggest that lamin–chromatin interactions dynamically regulate nuclear mechanics rather than serving solely as static structural linkages. Therefore, variations in lamin A/C–chromatin interaction strength determine NE mechanical stability and influence cellular adaptation to external stimuli by remodeling intranuclear stress distributions.

### 3.3 Physiological and Pathological Applications of Nuclear Mechanics Measurement Techniques

The 3D magnetic twisting cytometry (3D‐MTC) is a high‐resolution magnetic technique for quantitatively analyzing cellular mechanical properties and load‐bearing mechanisms [8]. In this method, magnetic fields applied at the cell surface are used to monitor rotational shear stress in real time. The 3D‐MTC system generates independently controlled magnetic fields using three coil pairs aligned along the *X*‐, *Y*‐, and *Z*‐axes [8]. First, a strong magnetic field (∼2,500G) was applied to magnetize the bead along a defined direction, followed by an orthogonal torsional sinusoidal field (approximately 0–25G) [8].

The spherical magnetic bead rotates within the plane formed between the two magnetic fields, transferring shear stress through its adhesive contact with the cell [8]. Quantifying bead‐center displacement enables the determination of cellular resistance to applied stress and derivation of effective cellular stiffness and viscoelastic properties. Consequently, 3D‐MTC serves as a precise technique for quantitatively measuring mechanical stress transmission through cell‐surface adhesions and intracellular load‐bearing pathways [8]. This technology enables real‐time monitoring of cellular stiffness and facilitates investigation of force transmission pathways connecting the cytoskeleton to the nucleus, providing a quantitative framework for mechanobiology research.

Among these approaches, dual OTs serve as high‐precision nanomechanical tools that quantitatively measure the mechanical properties of isolated nuclei through optical force manipulation of microscopic particles. OTs capture microscopic particles through optical gradient forces generated when a high‐power infrared laser passes through a high‐refractive‐index medium [120]. Near the laser focal point, the light‐intensity gradient pulls particles into an optical potential well, enabling stable 3D trapping and manipulation [120].

In a dual‐OT system, a polarization beam‐splitter cube divides a single laser beam into two independent optical traps. Each trap is focused on a PLL‐coated polystyrene bead attached to opposite sides of the cell nucleus [120]. These beads function as optical handles that pull or oscillate the nucleus, enabling precise mechanical stimulation of the entire nuclear structure [120]. Two PLL‐coated beads positioned on opposite sides of the isolated nucleus are independently controlled using optical traps [120]. Gradual increases in trap separation apply forces reaching hundreds of pN, while nuclear deformation and mechanical recovery are monitored in real time [120]. This approach generates a nonlinear force–extension curve for analyzing the nonlinear elastic behavior of the nucleus.

Additionally, the OT system supports active microfluidic control. Nuclear viscoelastic responses are measured using one bead, while the second bead oscillates at small amplitudes over a frequency range of 0.01–100 Hz under a defined nuclear prestress [120]. This approach enables accurate characterization of frequency‐dependent nuclear mechanics by simultaneously estimating time scale‐dependent elastic and viscous moduli.

In cancer cells, changes in nuclear mechanical dynamics are closely associated with chromatin compaction and decompaction. While chromatin compaction generally increases nuclear stiffness by reducing deformability, acute chromatin decompaction can paradoxically increase nuclear stiffness over short timescales through nuclear swelling and enhanced nuclear import [121]. However, in cancer cells, the association between chromatin decompaction and increased nuclear stiffness progressively weakens with increasing malignancy [121]. Low‐ and high‐malignancy breast cancer cell lines (MCF‐7 and MDA‐MB‐231) exhibit distinct nuclear size and mobility patterns associated with chromatin organization and histone post‐translational modifications [121]. Therefore, increasing research effort focuses on exploiting nuclear mechanical properties as diagnostic biomarkers for cancer.

Conventional AFM‐based cellular mechanical analyses rely primarily on cell‐surface indentation to indirectly infer perinuclear mechanical properties or to measure nuclei isolated from cells. Nanoendoscopy‐AFM (NE‐AFM) is an AFM‐based technique designed to directly and precisely measure nuclear mechanical properties, particularly elasticity, in living cells (Figure 4A) [33]. The core component of NE‐AFM was a nano‐needle probe mounted on an AFM cantilever, repeatedly inserted into the cell interior. Experimental parameters, including probe geometry, insertion depth, and applied force, were optimized to minimize cellular damage and preserve viability during repeated insertions [33].

FIGURE 4Probing mechanical regulation of the nuclear envelope and chromatin. (A) Conceptual schematic of nanoendoscopy–AFM. An ultra‐fine nanoneedle probe (≈160 nm diameter, ≈24 nm tip radius) mounted on an AFM cantilever is positioned above a living cell. The probe penetrates the plasma membrane and cytoplasm and advances along a defined trajectory toward the nucleus. Cytoplasmic and nuclear regions are indicated in green and blue, respectively. F–z curves are recorded at the cell CP during membrane engagement and at the nuclear CP during nuclear indentation, enabling mechanical characterization of both cytoplasm and nucleus. Adapted with permission [33] 2025, *ACS Applied Nano Materials*. (B) Conceptual schematic of direct nuclear force probing using a micropipette. A glass micropipette (≈0.5 μm diameter) is sealed onto the nuclear surface using suction pressure. A localized tensile force is applied directly to the nucleus by translating the micropipette away from the nucleus at constant velocity while maintaining suction. This configuration enables direct nuclear loading without involving cytoplasmic structures or cell–substrate adhesions. Bottom: after pipette attachment, a pulling force of ∼6 nN induces progressive nucleus elongation and displacement along the direction of pipette movement. Top: Adapted with permission [34] 2015, *Proceedings of the National Academy of Sciences*. Bottom: Adapted with permission [122] 2018, *Journal of Visualized Experiments*. (C) Conceptual schematic of single‐pole magnetic tweezers. A ferromagnetic bead internalized via endocytosis is positioned near the nucleus using a magnetic pole tip. A magnetic field gradient applies F to the bead, inducing nuclear deformation, which is monitored in real time. NE wrinkle profiles and local curvature are quantified using R. Adapted with permission [35] 2023, *Proceedings of the National Academy of Sciences*. (D) Conceptual schematic of modified magnetic torque tweezers. Torsionally constrained DNA handles tether nucleosome arrays between a functionalized surface and a superparamagnetic bead, enabling application of controlled tension and torque via external magnet translation and rotation. Increasing force and twist progressively unwraps chromatin, exposing DNA templates. Bead position and rotation tracked with high‐magnification microscopy are used to quantify tether extension and torsional response. Adapted with permission [123] 2020, *Nature Communications*. (E) Conceptual schematic of single‐molecule optical tweezers–fluorescence assay. Dual‐trap optical tweezers capture streptavidin‐coated beads and apply precise tensile forces (1–15 pN) to DNA or chromatin tethers. Nucleosome arrays are connected via DNA handles. Simultaneous measurements of tether extension and fluorescence signals allow direct correlation of nucleosome unwrapping events with fluorescence changes along the construct. Adapted with permission [36] 2022, *Nature Communications*. (F) Conceptual schematic of FRET‐based nuclear lamina tension sensor. The Lamin‐SS sensor comprises a FRET module flanked by two lamin A/C‐specific nanobodies that bind distinct lamin A/C molecules. The donor (mTFP) and acceptor (mVenus) fluorophores are connected via an elastic peptide linker. FRET efficiency reflects the distance between mTFP and mVenus, reporting lamina tension. Adapted with permission [14] 2023, *Nature Communications*. AFM, atomic force microscopy; CP, contact point; F, force; F–z, force–distance; Lamin‐SS, simple spring; mTFP, monomeric teal fluorescent protein; mVenus, monomeric Venus fluorescent protein; NE, nuclear envelope; R, radius of curvature.

**Figure:**
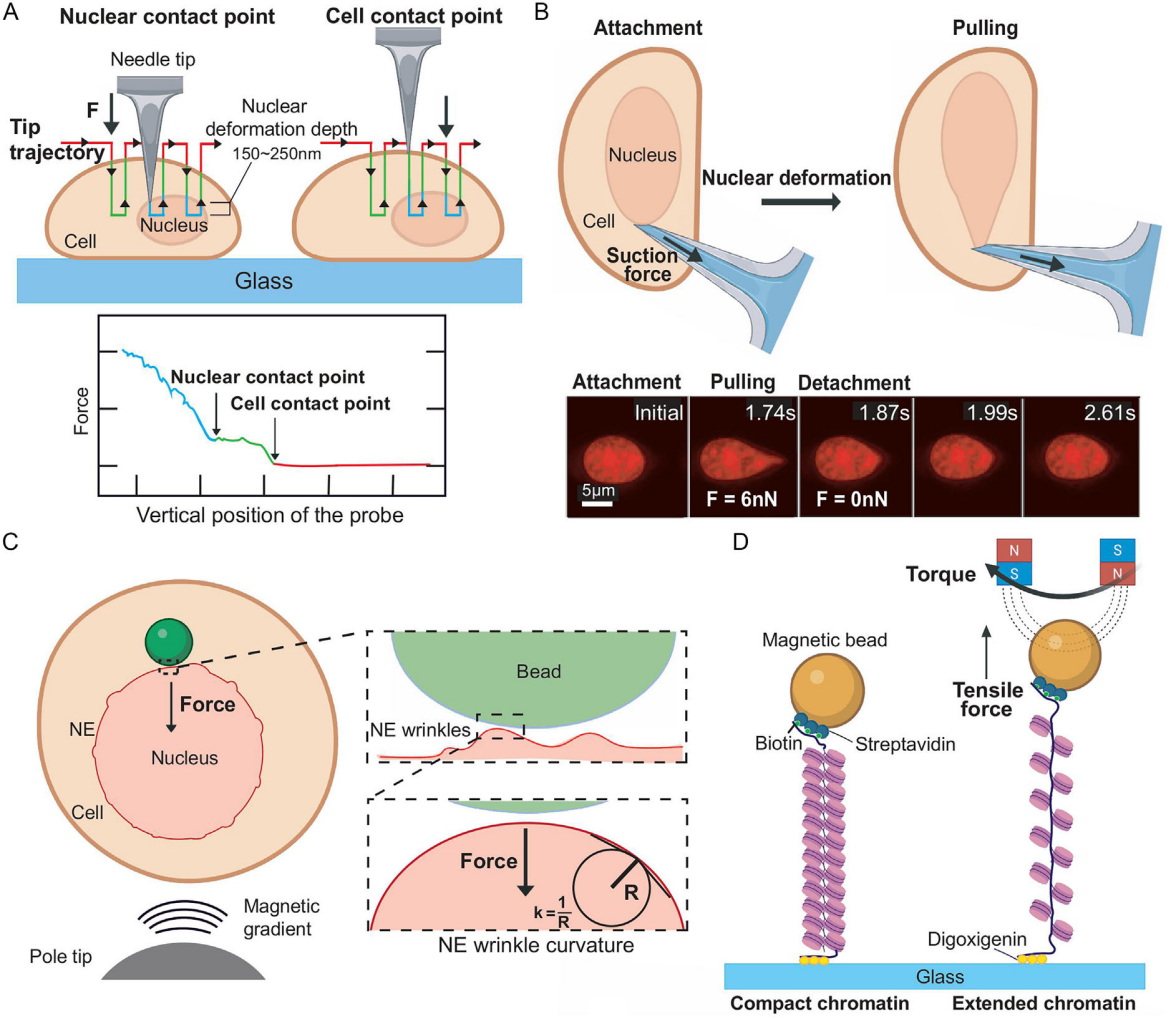

**Figure:**
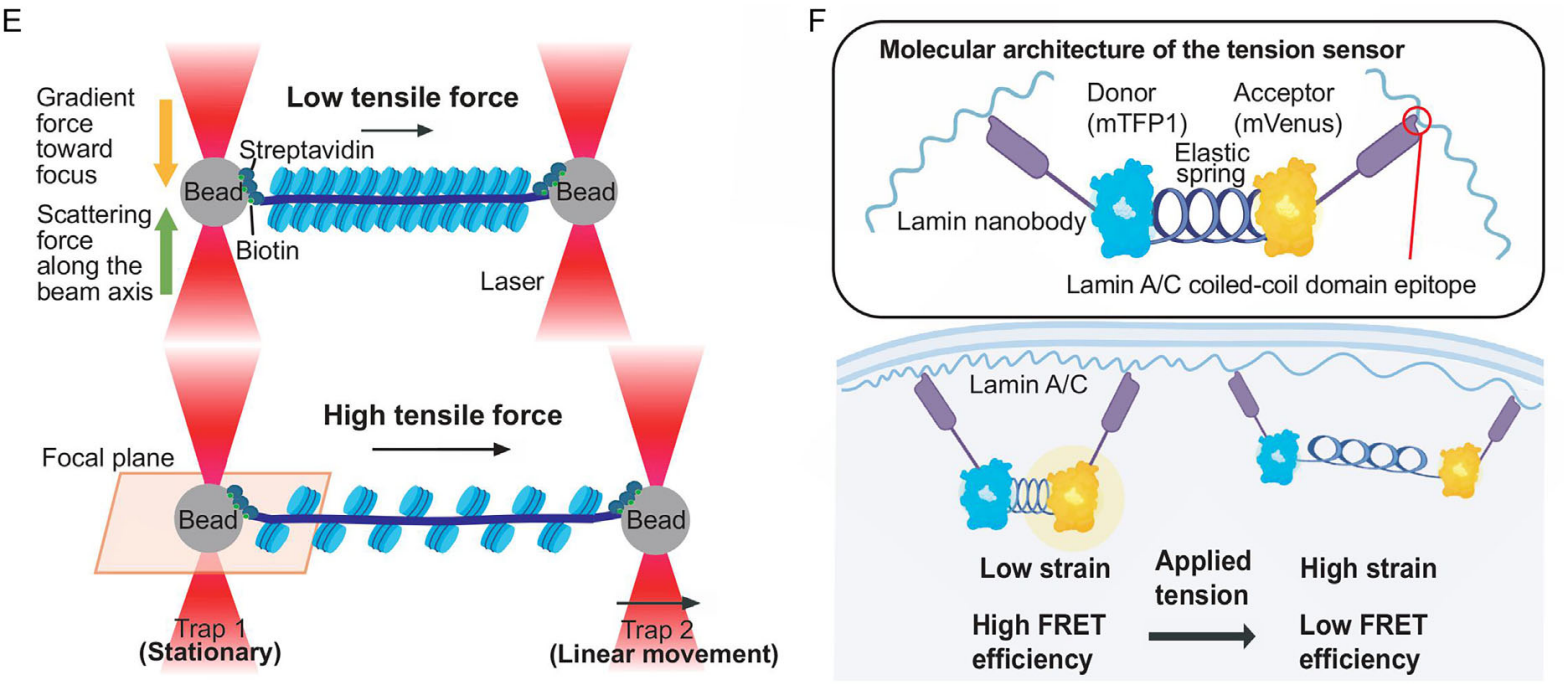

Measurements were based on force–distance (F–z) curves. The initial probe–membrane interaction defines the cell contact point (Figure 4A) [33]. Beyond this point, cantilever deflection is monitored through laser reflection changes to calculate the vertical force exerted by the nucleus on the probe [33]. Upon nuclear contact after cytoplasmic traversal, the force signal increases sharply, indicating the onset of nuclear mechanical resistance (Figure 4A) [33]. A shallow indentation depth of approximately 150–250 nm was applied to measure the elastic response of nuclear structures, including the NE and chromatin [33].

During indentation, vertical probe displacement (*z*) and force (*F*) were recorded to generate F–z curves. Indentation data from the F–z curves were fitted to a mechanical model, such as a modified Hertz model, to calculate Young's modulus [33]. These values were integrated into an elasticity map to visualize nuclear mechanical heterogeneity [33]. NE‐AFM is used to detect changes in nuclear elasticity in cancer cells. PC9 cells—a primary lung cancer cell line—exhibit nuclear elasticity values of 2714 ± 126 Pa under serum‐containing conditions and 4384 ± 284 Pa under serum deprivation, indicating a significantly increased elasticity during serum deprivation [33]. Increased stiffness is attributed to enhanced chromatin condensation, as immunoblotting shows a significant 3.22 ± 1.76‐fold increase in H4K20me3 levels under serum‐deprived conditions [33]. Serum deprivation enhances chromatin condensation in cancer cells, resulting in increased nuclear stiffness.

Conversely, TGF‐β‐induced EMT significantly reduces nuclear stiffness compared to control cells [33]. Given that cell membrane elasticity remains unchanged after TGF‐β treatment, the reduction in nuclear stiffness is attributed to EMT‐associated intranuclear remodeling, including altered chromatin condensation, rather than plasma membrane changes. Therefore, NE‐AFM extends beyond cancer progression to nuclear mechanical reprogramming across diverse physiological and pathological contexts.

Under pathological conditions, fibroblasts differentiate into contractile myofibroblasts in response to persistent mechanical and inflammatory stimuli. They acquire actin stress fibers, deposit excess ECM, and promote progressive tissue stiffening, thereby reinforcing fibrotic remodeling [124]. Concurrent changes in cellular and nuclear mechanics, together with chromatin reorganization, maintain pro‐fibrotic gene expression and drive disease progression.

Chromatin compaction and histone modifications directly regulate nuclear stiffness and mechanotransduction. In highly condensed chromatin states, the DNA–histone complex is densely packed, increasing nuclear viscoelasticity and resistance to external deformation [125]. This state promotes ECM accumulation and fibrotic responses. In contrast, histone acetylation‐driven chromatin decompaction softens nuclear architecture, increases nuclear deformability, and suppresses pathological fibroblast activation [125]. Moreover, micropipette‐based force probing enables quantification of nuclear elastic and viscoelastic properties by directly measuring deformation responses. Micropipette assays use glass micropipettes with diameters of several micrometers coupled to a precise pressure control system (Figure 4B) [34].

Nuclear mechanics were quantified using three micropipette deformation assay‐derived key parameters. First, aspiration‐induced nuclear deformation was quantified as length strain (*ε*) [34]. This parameter indicates the degree of nuclear stretching during aspiration or traction, representing the immediate nuclear mechanical response. Second, aspiration‐induced nuclear displacement was quantified as the distance traveled by the posterior nuclear edge [34]. This metric reflects nuclear mobility under external force and serves as an indirect measure of mechanical coupling between the nucleus and the surrounding environment. Recovery dynamics were analyzed by monitoring nuclear behavior following force release (Figure 4B) [34, 122]. The rate and extent of nuclear shape and positional recovery were quantified to differentiate the elastic and viscous components of nuclear mechanics [34]. This approach enables the characterization of nuclear mechanical properties based on time‐dependent deformation responses.

In NIH3T3 cells, micropipette‐applied force induced measurable nuclear deformation at low suction pressures, with deformation increasing with suction pressure [34]. Nuclear deformation was quantified as length strain (*ε* = *L*/*L*_0_ − 1), which increased proportionally with applied tensile force [34]. Nuclear deformation consistently exceeded nuclear displacement [34]. These findings indicate strong mechanical coupling between the nucleus and the surrounding cellular structure, with nuclear deformation occurring preferentially to nuclear translation under external force. After force removal, the nucleus rapidly recovered its shape. The nuclear length strain relaxed within several milliseconds, indicating high elastic resilience [34]. In contrast, positional recovery was significantly slower than shape recovery, suggesting distinct mechanisms for shape deformation and positional displacement [34].

Vimentin‐deficient cells exhibit increased nuclear deformation (21 ± 3% to 38 ± 4%) and nuclear translation (1.74 ± 0.35 to 2.89 ± 0.57 μm) [34]. The increase in nuclear deformation indicates that vimentin intermediate filaments are major cytoskeletal components resisting nuclear deformation. In vimentin‐deficient cells, the nuclear deformation recovery rate remained unchanged or increased slightly (69 ± 4% to 76 ± 2%), whereas the nuclear positional recovery rate decreased significantly (121 ± 14% to 72 ± 5%) [34]. Collectively, these findings indicate a mechanical hierarchy within the nucleus: elastic shape recovery is rapid and largely independent of the cytoskeletal context, while translational repositioning depends on vimentin intermediate filaments. This distinction highlights the different mechanical roles of nuclear–cytoskeletal coupling in 3D environments. Increased nuclear stiffness and altered recovery dynamics in activated fibroblasts and myofibroblasts serve as functional indicators of fibrosis progression.

A 3D‐MTC assay was used to quantify nuclear stiffness from chromatin‐mediated deformation. In this technique, localized shear stress was applied to magnetic beads bound to cell‐surface integrin receptors. Force transmission across the cytoskeleton induced nuclear deformation [8]. First, magnetic beads (∼4 μm in diameter) were uniformly coated with RGD peptide to facilitate specific integrin‐mediated attachment [8]. Following magnetization with a strong magnetic pulse (2,500G) along the *Z*‐axis, a weak alternating magnetic field was applied along the *X*‐ or *Y*‐axis to rotate the bead and generate a constant‐amplitude sinusoidal shear stress (15 Pa) on the attached cell [8]. The applied stress was transmitted across the cytoskeleton, inducing deformation of intranuclear chromatin.

Real‐time displacement analysis of green fluorescent protein (GFP)‐labeled loci at the bacterial artificial chromosome insertion site shows that chromatin mean square displacement increases with stress angle, reaching a maximum at 90° and a minimum at 0° [8]. These findings indicate directional transmission of externally applied stress to chromatin. Chromatin elongation along the 90° axis is approximately threefold greater (∼18%) than that along the 0° axis [8]. Local deformation rate mapping shows that chromatin tensile strain is approximately 2.5‐fold higher than the shear strain [8]. This finding indicates that local cell‐surface shear stress is primarily converted to tensile deformation within the nucleus. Therefore, nuclear mechanical responses depend on the magnitude of the applied stress and the anisotropic properties associated with stress direction and cytoskeletal organization. To evaluate the effect of chromatin deformation on nuclear stiffness, the Δ displacement between two GFP‐labeled loci at each stress angle was compared to cell stiffness values [8]. Chromatin deformation was inversely related to cell stiffness [8]. Lower cell stiffness was associated with greater chromatin deformation, suggesting that reduced cytoskeletal tension permits deeper transmission of external forces to the nucleus and promotes local nuclear softening. Overall, chromatin‐based nuclear mechanotransduction analysis using 3D‐MTC provides a framework for understanding fibrosis as excessive ECM accumulation and dysregulated cell–nuclear mechanosignaling.

Sustained inflammation drives persistent changes in cellular signaling and mechanics, contributing to chronic diseases, such as fibrosis, cancer, and age‐related pathologies [126]. Inflammatory responses are regulated at the signaling and transcriptional levels, alongside structural remodeling of the nucleus and reorganization of cellular mechanical properties. Inflammatory cytokines (e.g., TNF‐α and IL‐1β) and reactive oxygen species reduce Lamin A/C expression and phosphorylation, increasing nuclear fragility and promoting NE rupture, DNA damage, and secondary inflammatory signaling, ultimately compromising cellular mechanical stability [127]. In contrast, inflammation‐induced chromatin remodeling is closely associated with the nuclear mechanical environment. Inflammation‐related transcription factors, including nuclear factor κB and signal transducer and activator of transcription 3, enhance immune‐related gene expression via histone modifications regulating chromatin accessibility [126]. Inflammatory responses involve coordinated activation of cell‐surface receptors and concomitant changes in cytoskeletal organization and nuclear mechanics.

Single‐pole MTs generated direct mechanical forces on the nuclei in living cells, enabling real‐time measurement of nuclear deformation and stiffness changes (Figure 4C) [35]. Magnetic field gradients applied force to magnetic beads, enabling precise, noncontact application of piconewton‐scale forces [35]. The single‐pole configuration generates a strong unidirectional magnetic gradient, applying directionally defined forces to individual magnetic beads within the cell [35]. Magnetic field application displaces the beads along the gradient direction [35], transmitting stress directly to the nucleus and inducing nuclear deformation. Nuclear displacement was quantified using real‐time image analysis to derive the force–displacement relationship [35]. The nuclear mechanical response was assessed using viscoelastic modeling of force–displacement data, confirming nuclear stiffening.

To define the structural basis of these mechanical changes, fluorescence microscopy‐based NE imaging was combined with a curvature‐based analytical metric (Figure 4C) [35]. NE morphology was visualized using the NE marker LAP2β–red fluorescent protein (Lap2β‐RFP), while force‐induced changes in envelope folding were quantitatively analyzed [35]. Average curvature (κ) was used as a quantitative measure of NE deformation in the locally stressed region [35]. During the pre‐cycle phase without applied external force, the average curvature at the indentation site was 0.08 µm^−1^. Periodic force application progressively reduced NE curvature across successive cycles (0.08 → 0.05 → −0.10 → −0.16 → −0.18 µm^−1^) [35]. These findings suggest that NE unfolding accumulates progressively under repeated mechanical stimulation rather than requiring a single critical force threshold.

Quantitative analysis of the NE unfolding−nuclear stiffness relationship shows that nuclei with greater curvature reduction (cycles 1−4) exhibit higher stiffening ratios [35]. For example, a nucleus with minimal curvature reduction (0.01 µm^−1^) exhibits a low stiffening ratio (1.06), while a nucleus with substantial curvature reduction (0.42 µm^−1^) shows a high stiffening ratio (1.49) [35]. These findings indicate that greater NE wrinkle unfolding correlates with increased nuclear stiffness. FEM simulations further demonstrate that NE wrinkle unfolding alone increases the effective nuclear stiffness [35].

Mechanical transitions may occur in pathological environments associated with chronic inflammation or persistent tissue injury. In these microenvironments, cells experience sustained mechanical stress, ECM remodeling, and altered physical interactions with neighboring cells [126]. Under these conditions, repeated NE unfolding without structural recovery may maintain nuclear stiffening and contribute to delayed inflammatory resolution or chronic inflammation. Therefore, NE folds may serve as key mechanical regulators of cellular function and tissue homeostasis in inflammatory environments rather than serving solely as morphological features. This insight could inform the development of strategies to modulate nuclear mechanical states or use them as diagnostic markers for future inflammation‐related diseases.

Cellular aging is also associated with global reorganization of gene expression and postnatal genomic changes, along with changes in chromatin accessibility and nuclear mechanical properties. Dysregulation of NE proteins and LINC complexes increases nuclear stiffness and mechanical sensitivity in aged cells [128]. ATAC‐seq analysis of neonatal and senescent human dermal fibroblasts was performed to assess genome‐wide chromatin accessibility [128]. The analysis reveals 18,377 neonatal‐specific open chromatin regions, 39,611 senescence‐specific regions, and approximately 74,736 shared regions [128]. These findings indicate that aging is associated with extensive chromatin reorganization.

An MT system was used to precisely control and measure forces and torques applied to individual chromatin fibers (Figure 4D). Chromatin fibers were generated by reconstituting histone octamers onto repetitive DNA sequences and contained two distinct nucleosome repeat lengths (NRLs) [123]. Nucleosome‐free DNA handle segments were incorporated at both ends of the chromatin fiber. One end was anchored to a glass surface, while the other was attached to a superparamagnetic bead (Figure 4D) [123]. This tether configuration stably immobilized individual chromatin fibers, enabling direct transmission of externally applied mechanical forces. The MT system rotationally constrained the chromatin fiber, allowing rotation‐induced twist changes to accumulate within the fiber [123]. This configuration enabled quantitative analysis of intrinsic torsional elasticity and twist coefficients [123].

Force magnitude was controlled by adjusting the vertical position of the magnet pair. Moving the magnets closer to the bead increased the magnetic field gradient, producing a tensile force on the chromatin fiber [123]. This force was maintained within the pN range, and fiber extension was quantified via bead displacement along the *z*‐axis [123]. Torque was applied by rotating the magnet pair, inducing bead rotation and altering the twist of the DNA tether attached to the chromatin fiber. The resulting accumulated torsional stress enables quantitative characterization of the torsional resistance of the chromatin fiber [123].

Single‐molecule MTs were used to quantify the mechanical responses of 167–NRL and 197–NRL chromatin fibers under applied force and torque. Under rotational constraint, both fiber types adopted left‐handed superhelical conformations [123]. Under positive rotation, chromatin fibers absorbed substantial torque and delayed plectoneme formation in the DNA handles [123]. In the rotation–elongation curve, fiber length remains largely constant up to ∼25 turns and decreases only with further rotation [123]. This finding indicates that the chromatin fiber absorbs initial torsional stress rather than transmitting it to the DNA. Under negative rotation, chromatin fibers show minimal twist absorption, while torsional stress is transmitted directly to the DNA handle [123]. In this case, changes in extension reflect the torsional response of the DNA handle rather than structural rearrangements within the chromatin fiber [123]. Direct torque measurements further support this asymmetry. Under positive rotation, chromatin fibers exhibit gradual torque accumulation, with total torque remaining <10 pN·nm even after 30 rotations [123].

Furthermore, force–extension assays resolved the nucleosome unstacking transition. At forces <4 pN, 167–NRL chromatin fibers exhibit identical stretching curves under both rotational constraints [123]. However, differences were observed at the 4–6 pN force range [123]. The abrupt extension increase of ∼0.5 μm in this force range corresponds to the nucleosome unstacking transition, including nucleosome unstacking, linker DNA release, and DNA unwrapping of ∼56 bp per nucleosome [123]. Structural differences between NRLs were also observed. The 167–NRL fiber adopts a two‐start configuration, while the 197–NRL fiber forms a one‐start (solenoidal) structure; however, both exhibit high torsional absorption capacity [123]. This finding suggests that chromatin torsional elasticity is a general mechanical property of condensed chromatin fibers rather than a feature restricted to a specific higher‐order architecture. With aging, key determinants of chromatin structural stability, including lamin–nuclear skeleton interactions, nucleosome occupancy and spacing, histone modification patterns, and heterochromatin organization, are altered [128].

Shifts in mechanical parameters, such as chromatin fiber torsional elasticity, torque absorption capacity, and unstacking threshold, potentially accompany these age‐related changes. At the molecular level, these parameters may serve as functional indicators of intrinsic nuclear susceptibility to torsional stress associated with replication and transcription. The single‐molecule OT–fluorescence assay facilitates quantitative mechanical force application to individual chromatin molecules while simultaneously monitoring the real‐time spatiotemporal dynamics of proteins and histones [36]. This approach integrates dual‐trap OT with confocal fluorescence microscopy along a shared optical axis. Laser‐based OTs capture microbeads, with two independent traps anchoring both ends of the chromatin fiber [36]. Controlled adjustment of the inter‐trap distance enables stable application of pN‐range tensile forces to individual chromatin molecules (Figure 4E) [36].

Several‐kilobase DNA handles were attached to both ends of the chromatin array and linked to beads captured by OT via biotin–streptavidin interactions (Figure 4E) [36]. These DNA handles transmit force and serve as structural buffers, enabling precise control of the tension applied to the nucleosome array [36]. Fluorescent labeling enables visualization of protein and histone dynamics. Histones are labeled with a red fluorophore, while Plk1‐interacting checkpoint helicase (PICH) is expressed as an enhanced green fluorescent protein (eGFP) fusion protein [36]. Confocal fluorescence microscopy scans fluorescence signals along the DNA axis, enabling quantitative measurement of protein binding, molecular motion, and histone repositioning over time [36].

Under constant tensile force applied via OT, chromatin rearrangement or partial nucleosome unwrapping increases the effective DNA length, measured as increased bead‐to‐bead distance (Figure 4E) [36]. Simultaneous fluorescence tracking of PICH binding and motion directly links the mechanically induced activity to chromatin structural changes. This approach was used to quantitatively measure nucleosome loosening frequency, mean loosening duration, and force‐dependent changes in loosening probability [36]. The relationship between force and loosening frequency was fitted with an Arrhenius‐type model to estimate changes in the energy barrier governing nucleosome stability [36].

Quantitative analysis of PICH‐induced effects on nucleosome structure in tensioned chromatin shows that, under low tension (<3 pN), PICH primarily binds to nucleosome‐free DNA handle regions [36]. At forces >3 pN, PICH penetrates and binds within nucleosome arrays [36]. These findings indicate that PICH selectively associates with nucleosome arrays only above a mechanical tension threshold [36]. Nucleosome unwinding kinetics were quantified under force‐clamp conditions at constant forces (3–15 pN) [36]. Under high tension, the outer DNA turn is already unwound; therefore, stepwise increases in DNA length primarily reflect inner‐turn unwinding [36]. The observed step size is approximately 26 nm, consistent with unwinding of ∼80 bp of DNA from the inner turn [36].

In the presence of PICH and adenosine triphosphate (ATP), nucleosome unwinding frequency increases significantly across all force conditions, indicating that PICH promotes tension‐induced nucleosome unwinding [36]. Arrhenius‐type analysis of force‐dependent unwinding probability shows that PICH and ATP reduce the half‐force required for inner‐turn nucleosome unwinding from ∼10.6 pN to 5.5 pN [36]. These findings suggest that PICH acts as a potent chromatin remodeling factor that reduces the energy barrier for tension‐induced nucleosome unwinding by >50%. Simultaneous tracking of fluorescently labeled histones and PICH–eGFP confirms that histones remain DNA‐bound for a significant period after nucleosome unwinding rather than dissociating immediately [36]. Furthermore, histone sliding along DNA occurs in the presence of PICH and ATP, indicating that PICH promotes nucleosome disassembly and dynamically regulates chromatin accessibility by repositioning histones after disassembly [36]. Collectively, the integrated OT–fluorescence microscopy single‐molecule approach enables quantitative characterization of PICH binding, nucleosome unwinding, energy barrier changes, and histone dynamics under defined mechanical tension [36].

Chromatin serves as both a repository of genetic information and a dynamic mechanical system actively modulating its structure and energy landscape under mechanical tension [36]. Aged cells often exhibit chromosome segregation errors during mitosis, persistent chromatin bridges, and impaired resolution of DNA entanglements, associated with defective tension‐dependent chromatin remodeling [128]. Specifically, decreased activity or altered force‐response profiles of tension‐sensitive chromatin remodeling enzymes may enable early molecular detection of age‐related chromatin functional decline. This framework could be extended as a diagnostic strategy to quantify age‐related accumulation of nuclear structural and functional changes from a mechanical perspective.

Lamin A regulates chromatin epigenetic organization via LADs [129]. In lamin A/C‐deficient cells, heterochromatin formation at the nuclear periphery is impaired during muscle differentiation, disrupting muscle‐specific gene expression programs [130]. A study reports that lamin A/C deficiency fundamentally reorganizes 3D genome architecture from the embryonic stem cell stage onward. Consistent with these findings, LMNA‐deficient cells exhibit enlarged nuclei, irregular nuclear morphology, and enlarged nucleoli compared with control cells [130]. Three‐dimensional fluorescence in situ hybridization (FISH) analysis reveals 14 spatial extensions, suggesting that lamin A/C maintains a compressive organization in specific chromosomal regions [130]. These findings indicate that lamin A/C functions beyond a structural nuclear scaffold, mediating mechanical coupling between the NE and chromatin while regulating intranuclear mechanical tension, thereby contributing to laminopathy pathophysiology.

Physiological state‐dependent changes in mechanical tension on lamin A/C under different cellular conditions were quantitatively assessed using a FRET‐based Lamin‐SS sensor. Tension on lamin A/C was detected via an intramolecular FRET, consistent with standard FRET‐based sensors [14]. Two lamin A/C‐specific nanobodies were positioned on opposite sides of the FRET module (Figure 4F). Nanobody binding to lamin A/C changes the interprotein distance and modulates the FRET signal, enabling tension detection (Figure 4F) [14]. The nanobody exhibits high‐affinity binding to a defined epitope at the terminal region of the lamin A/C coiled‐coil domain [14]. This epitope mediates intermolecular interactions between adjacent lamin molecules during filament assembly. Nanobody‐mediated anchoring of the TSMod module localizes the sensor to regions of elevated mechanical stress within the lamin network [14]. This configuration enables real‐time monitoring of nuclear lamina responses to extracellular forces and intracellular mechanical cues.

Initial FRET efficiency measurements in cells expressing Lamin‐SS reveal substantial intercellular variability [14]. This variability suggests that cellular physiological factors, including cell‐cycle stage, cell morphology, and intranuclear chromatin organization, influence mechanical tension on lamin A/C. To test this hypothesis, key physiological processes associated with nuclear structural remodeling, including cell‐cycle arrest, EMT, and chromatin reorganization, were experimentally induced [14].

Specifically, early S‐phase arrest induced with the DNA polymerase α inhibitor aphidicolin significantly increases Lamin‐SS FRET efficiency (16%–21%) [14]. Given that increased FRET efficiency corresponds to reduced molecular tension, these findings indicate reduced mechanical force on lamin A/C during early S phase. However, persistent cell‐to‐cell variability in FRET values under identical experimental conditions further suggests that factors beyond cell‐cycle stage contribute to lamin tension heterogeneity [14].

Subsequently, lamin A/C tension was examined during TGF‐β‐induced EMT. EMT is associated with increased cell contractility and actin stress‐fiber formation; however, Lamin‐SS FRET efficiency increases from 16% to 22%, indicating reduced tension at the nuclear lamina [14]. Given that higher FRET efficiency indicates lower molecular tension, these findings suggest that increased cellular contractility coincides with reduced force transmission to the NE, likely due to reorganization of intracellular mechanical coupling and altered nucleus–cytoskeleton force transmission.

To evaluate the lamin tension–chromatin organization relationship, chromatin condensation states were pharmacologically induced [14]. Chromatin decondensation induced with a histone deacetylase inhibitor significantly increases Lamin‐SS FRET efficiency (15%–20%) [14], indicating reduced lamin A/C tension. Conversely, chromatin condensation induced with a histone demethylase inhibitor decreases FRET efficiency (17%–15%); however, this change is not statistically significant [14]. These findings indicate that chromatin condensation is associated with a modest increase in lamin tension, with an overall limited effect.

To determine the effects of chromatin state on lamin A/C structural organization, NE accessibility, and lamin filament organization were quantitatively analyzed using antibodies targeting distinct lamin A/C epitopes.^14^ Antibody‐labeling ratios show no significant change after trichostatin A or methylstat treatment, and fluorescence recovery after photobleaching analysis reveals unchanged Lamin‐SS binding dynamics [14]. These findings suggest that changes in FRET efficiency reflect altered transmission of chromatin state‐dependent nuclear lamina tension rather than lamin A/C structural rearrangement.

Overall, physiological factors, including cell cycle, EMT, and chromatin organization, regulate lamin A/C tension, with a general trend toward reduced nuclear lamina tension, particularly during chromatin decondensation. These findings indicate that chromatin compaction regulates mechanical tension on lamin A/C. Low FRET efficiency values occur only under physical compression, suggesting that NE deformation directly alters lamin A/C tension. The mechanical properties of lamin were quantified using a combined OT–fluorescence microscopy approach. The nuclear lamina and chromatin, the primary mechanical components of the nucleus, were labeled with different fluorophores (LaminB1–Alexa 488 and histone H3–Alexa Fluor 647 antibodies) [120].

During nuclear stretching, multichannel fluorescent images were acquired to track real‐time responses of lamin and chromatin to applied forces [120]. Both structures consistently elongate along the direction of the applied force under increased tension [120]. In contrast, lamin contracts perpendicular to the tensile axis (Poisson effect), suggesting that the lamin network dissipates stress through structural reorganization rather than uniform stretching [120]. Lamin area change is relatively small (<5%) compared with chromatin, and its force–response slope is approximately one‐fifth that of histones [120]. These findings indicate greater chromatin deformability than lamin under tensile loading.

Mechanical characterization of lamin during NE tether formation reveals thin filamentous structures in approximately 30% of nuclei, exhibiting a force plateau persisting over a defined extension range even at low forces (<10 pN/μm) [120]. These filaments exhibit lower elasticity and higher viscosity than the bulk nucleus, along with frequency‐dependent viscoelastic behavior [120]. Fluorescent staining shows no lamin signal in these structures, comprising primarily lipid bilayers, indicating derivation from the NE rather than the plasma membrane [120].

Overall, the lamin layer deforms minimally under external forces, dissipating stress through cross‐sectional contraction and structural reorganization [120]. Additionally, lamin accommodates deformation by forming flexible membrane‐derived structures while maintaining NE stability. These findings indicate that the lamin network contributes to nuclear rigidity, force transmission, and mechanical buffering.

## 4 Future Perspectives

Mechanobiological evidence indicates that defects in cellular mechanotransduction contribute to the onset and progression of diseases, including cancer, fibrosis, age‐related disorders, and laminopathies, offering a mechanistic framework for their pathogenesis. Mechanical imbalance disrupts cellular architecture, NE integrity, and gene regulation, ultimately promoting pathological tissue remodeling. Recent mechanobiology research has shifted focus from cellular force transmission to nuclear‐ and chromatin‐level mechanics. Mechanical cues from the ECM and cell–cell junctions are transmitted through the cytoskeleton to the nucleus and regulate transcription and gene expression. Quantitative force measurement techniques, including AFM, OT, MT, and FRET‐based mechanosensors, enable real‐time monitoring of force–response relationships at the cellular and nuclear levels.

Future research should integrate multiscale mechanical measurements to connect cellular adhesions and junctions with nuclear and chromatin mechanics. This integrative approach is essential for understanding the regulatory effects of external and internal forces on gene expression, chromatin organization, and cell fate determination. Combining biophysical measurements with computational modeling will enable precise interpretation of localized mechanical perturbations and their effects on cellular and nuclear structure. Targeting mechanotransduction pathways offers clear translational opportunities for therapeutic and diagnostic applications. Key strategies include identifying early‐stage biomarkers of mechanical dysregulation and developing pharmacological agents or biomaterials for restoring mechanical homeostasis. These strategies are particularly relevant for mechanically sensitive diseases, including cancer, fibrosis, age‐related disorders, and laminopathies, by addressing the underlying mechanical disturbances. Ultimately, integrating mechanical information across molecular, cellular, and tissue scales and clarifying the mechanogenomic interaction principles remains a central challenge in mechanobiology research.

## Author Contributions

S.K., J.L, and K.L. co‐wrote the manuscript. D.K. supervised the project and contributed to manuscript writing.

## Funding

D.K. was supported by the National Research Foundation of Korea (RS‐2024‐00507251, RS‐2023‐00221182, and 26V0500‐26‐P020). This work was also partially supported by the ICT Creative Consilience Program through the Institute of Information & Communications Technology Planning & Evaluation (IITP), funded by the Ministry of Science and ICT (MSIT), Republic of Korea (IITP‐2026‐RS‐2020‐II201819).

## Conflicts of Interest

The authors declare no conflicts of interest.

## Data Availability

All data required to evaluate the conclusions of this study are included in the main text.

## References

[1] D. W. Zhou , M. A. Fernández‐Yagüe , E. N. Holland , et al., “Force‐FAK Signaling Coupling at Individual Focal Adhesions Coordinates Mechanosensing and Microtissue Repair,” Nature Communications 12 (2021): 2359. 10.1038/s41467-021-22602-5 PMC8060400 33883558

[2] Y. Zheng , M. K. L. Han , R. Zhao , et al., “Optoregulated Force Application to Cellular Receptors Using Molecular Motors,” Nature Communications 12 (2021): 3580. 10.1038/s41467-021-23815-4 PMC8196032 34117256

[3] J. M. Kalappurakkal , A. A. Anilkumar , C. Patra , T. S. van Zanten , M. P. Sheetz , and S. Mayor , “Integrin Mechano‐Chemical Signaling Generates Plasma Membrane Nanodomains that Promote Cell Spreading,” Cell 177 (2019): 1738–1756.e1723. 31104842 10.1016/j.cell.2019.04.037 PMC6879320

[4] W. Zhang , C.‐H. Lu , M. L. Nakamoto , et al., “Curved Adhesions Mediate Cell Attachment to Soft Matrix Fibres in Three Dimensions,” Nature Cell Biology 25 (2023): 1453–1464. 37770566 10.1038/s41556-023-01238-1 PMC10567576

[5] S. J. Tan , A. C. Chang , S. M. Anderson , et al., “Regulation and Dynamics of Force Transmission at Individual Cell‐Matrix Adhesion Bonds,” Science Advances 6 (2020): eaax0317. 32440534 10.1126/sciadv.aax0317 PMC7228748

[6] M. Rübsam , A. F. Mertz , A. Kubo , et al., “E‐Cadherin Integrates Mechanotransduction and EGFR Signaling to Control Junctional Tissue Polarization and Tight Junction Positioning,” Nature Communications 8 (2017): 1250. 10.1038/s41467-017-01170-7 PMC5665913 29093447

[7] R. M. Houtekamer , M. C. van der Net , M. J. Vliem , et al., “E‐Cadherin Mechanotransduction Activates EGFR‐ERK Signaling in Epithelial Monolayers by Inducing ADAM‐Mediated Ligand Shedding,” Science Signaling 18 (2025): eadr7926. 40359261 10.1126/scisignal.adr7926

[8] A. Tajik , Y. Zhang , F. Wei , et al., “Transcription Upregulation via Force‐Induced Direct Stretching of Chromatin,” Nature Materials 15 (2016): 1287–1296. 27548707 10.1038/nmat4729 PMC5121013

[9] S. M. Schreiner , P. K. Koo , Y. Zhao , S. G. Mochrie , and M. C. King , “The Tethering of Chromatin to the Nuclear Envelope Supports Nuclear Mechanics,” Nature Communications 6 (2015): 7159. 10.1038/ncomms8159 PMC4490570 26074052

[10] T. Hurtado de Mendoza , E. S. Mose , G. P. Botta , et al., “Tumor‐Penetrating Therapy for β5 Integrin‐Rich Pancreas Cancer,” Nature Communications 12 (2021): 1541. 10.1038/s41467-021-21858-1 PMC7943581 33750829

[11] F. Alisafaei , D. Shakiba , Y. Hong , et al., “Tension Anisotropy Drives Fibroblast Phenotypic Transition by Self‐Reinforcing Cell–extracellular Matrix Mechanical Feedback,” Nature Materials 24 (2025): 955–965. 40128624 10.1038/s41563-025-02162-5 PMC12368865

[12] J. Liu , C. Zhao , X. Xiao , et al., “Endothelial Discoidin Domain Receptor 1 senses Flow to Modulate YAP Activation,” Nature Communications 14 (2023): 6457. 10.1038/s41467-023-42341-z PMC10576099 37833282

[13] H. Tsui , S. J. van Kampen , S. J. Han , et al., “Desmosomal Protein Degradation as an Underlying cause of Arrhythmogenic Cardiomyopathy,” Science Translational Medicine 15 (2023): eadd4248. 36947592 10.1126/scitranslmed.add4248

[14] B. E. Danielsson , B. George Abraham , E. Mäntylä , et al., “Nuclear Lamina Strain States Revealed by Intermolecular Force Biosensor,” Nature Communications 14 (2023): 3867. 10.1038/s41467-023-39563-6 PMC10313699 37391402

[15] X. Wang , P. Wang , L. Zhang , et al., “Mammalian Cells Measure the Extracellular Matrix Area and Respond through Switching the Adhesion State,” Nature Communications 16 (2025): 6870. 10.1038/s41467-025-62153-7 PMC12297719 40715114

[16] D. Kim and D.‐H. Kim , “Subcellular Mechano‐Regulation of Cell Migration in Confined Extracellular Microenvironment,” Biophysics Reviews 4 (2023): 041305. 38505424 10.1063/5.0185377 PMC10903498

[17] P. Zhuo , Q. Li , B. Yang , N. Li , Z. Luo , and F. Zhang , “Interaction of Integrin αvβ3 and Fibronectin under Fluid Shear Forces: Implications for Tumor Cell Adhesion and Migration,” Frontiers in Cell and Developmental Biology 13 (2025): 1512672. 40070879 10.3389/fcell.2025.1512672 PMC11894259

[18] Y.‐C. Yeh , J.‐Y. Ling , W.‐C. Chen , H.‐H. Lin , and M.‐J. Tang , “Mechanotransduction of Matrix Stiffness in Regulation of Focal Adhesion Size and Number: Reciprocal Regulation of Caveolin‐1 and β1 Integrin,” Scientific Reports 7 (2017): 15008. 29118431 10.1038/s41598-017-14932-6 PMC5678369

[19] G.‐Y. Lai , Y.‐C. Lee , H.‐J. Weng , et al., “Discoidin Domain Receptor Inhibitor DDR1‐IN‐1 Induces Autophagy and Necroptotic Cell Death in malignant Peripheral Nerve Sheath Tumor,” Cell Death Discovery 11 (2025): 83. 40025071 10.1038/s41420-025-02367-2 PMC11873111

[20] C. M. Borza , G. Bolas , X. Zhang , et al., “The Collagen Receptor Discoidin Domain Receptor 1b Enhances Integrin β1‐Mediated Cell Migration by Interacting with Talin and Promoting Rac1 Activation,” Frontiers in Cell and Developmental Biology 10 (2022): 836797. 35309920 10.3389/fcell.2022.836797 PMC8928223

[21] Y. Wang , N. A. Jerome , A. J. Kelleher , et al., “TRIM29 Promotes Bladder Cancer Invasion by Regulating the Intermediate Filament Network and Focal Adhesion,” Oncogene 44 (2025): 4047–4057. 40908312 10.1038/s41388-025-03557-z PMC12518127

[22] M. L. Cagigas , N. S. Bryce , N. Ariotti , S. Brayford , P. W. Gunning , and E. C. Hardeman , “Correlative Cryo‐ET Identifies Actin/Tropomyosin Filaments that Mediate Cell–substrate Adhesion in Cancer Cells and Mechanosensitivity of Cell Proliferation,” Nature Materials 21 (2022): 120–128. 34518666 10.1038/s41563-021-01087-z

[23] A. Byron , J. A. Askari , J. D. Humphries , et al., “A Proteomic Approach Reveals Integrin Activation State‐Dependent Control of Microtubule Cortical Targeting,” Nature Communications 6 (2015): 6135. 10.1038/ncomms7135 PMC4317495 25609142

[24] N. B. M. Rafiq , Y. Nishimura , S. V. Plotnikov , et al., “A Mechano‐Signalling Network Linking Microtubules, Myosin IIA Filaments and Integrin‐Based Adhesions,” Nature Materials 18 (2019): 638–649. 31114072 10.1038/s41563-019-0371-y

[25] J. Grolleman , N. C. A. van Engeland , M. Raza , et al., “Environmental Stiffness Restores Mechanical Homeostasis in Vimentin‐Depleted Cells,” Scientific Reports 13 (2023): 18374. 37884575 10.1038/s41598-023-44835-8 PMC10603057

[26] K. Duś‐Szachniewicz , S. Drobczyński , M. Woźniak , et al., “Differentiation of Single Lymphoma Primary Cells and Normal B‐Cells Based on Their Adhesion to Mesenchymal Stromal Cells in Optical Tweezers,” Scientific Reports 9 (2019): 9885. 31285461 10.1038/s41598-019-46086-y PMC6614388

[27] R. Ungai‐Salánki , E. Haty , T. Gerecsei , et al., “Single‐Cell Adhesion Strength and Contact Density Drops in the M Phase of Cancer Cells,” Scientific Reports 11 (2021): 18500. 34531409 10.1038/s41598-021-97734-1 PMC8445979

[28] X. Wang and T. Ha , “Defining Single Molecular Forces Required to Activate Integrin and Notch Signaling,” Science 340 (2013): 991–994. 23704575 10.1126/science.1231041 PMC3710701

[29] W. Wang , W. Chen , C. Wu , et al., “Hydrogel‐Based Molecular Tension Fluorescence Microscopy for Investigating Receptor‐Mediated Rigidity Sensing,” Nature Methods 20 (2023): 1780–1789. 37798478 10.1038/s41592-023-02037-0

[30] M. R. Pawlak , A. T. Smiley , M. P. Ramirez , et al., “RAD‐TGTs: High‐Throughput Measurement of Cellular Mechanotype via Rupture and Delivery of DNA Tension Probes,” Nature Communications 14 (2023): 2468. 10.1038/s41467-023-38157-6 PMC10147940 37117218

[31] I. Lüchtefeld , I. V. Pivkin , L. Gardini , et al., “Dissecting Cell Membrane Tension Dynamics and Its Effect on Piezo1‐Mediated Cellular Mechanosensitivity Using Force‐Controlled Nanopipettes,” Nature Methods 21 (2024): 1063–1073. 38802520 10.1038/s41592-024-02277-8 PMC11166569

[32] T. Kanadome , N. Hoshino , T. Nagai , T. Matsuda , and T. Yagi , “Development of FRET‐Based Indicators for Visualizing Homophilic Trans Interaction of a Clustered Protocadherin,” Scientific Reports 11 (2021): 22237. 34782670 10.1038/s41598-021-01481-2 PMC8593154

[33] T. Ichikawa , Y. Kono , M. Kudo , et al., “Probing Nanomechanics by Direct Indentation Using Nanoendoscopy‐AFM Reveals the Nuclear Elasticity Transition in Cancer Cells,” ACS Applied Nano Materials 8 (2025): 20239–20249. 41164208 10.1021/acsanm.5c03044 PMC12560078

[34] S. Neelam , T. J. Chancellor , Y. Li , et al., “Direct Force Probe Reveals the Mechanics of Nuclear Homeostasis in the Mammalian Cell,” Proceedings of the National Academy of Sciences 112 (2015): 5720–5725. 10.1073/pnas.1502111112 PMC4426403 25901323

[35] W. Tang , X. Chen , X. Wang , et al., “Indentation Induces Instantaneous Nuclear Stiffening and Unfolding of Nuclear Envelope Wrinkles,” Proceedings of the National Academy of Sciences 120 (2023): e2307356120. 10.1073/pnas.2307356120 PMC10483616 37639585

[36] D. Spakman , T. V. M. Clement , A. S. Biebricher , et al., “PICH Acts as a Force‐Dependent Nucleosome Remodeler,” Nature Communications 13 (2022): 7277. 10.1038/s41467-022-35040-8 PMC9700735 36433994

[37] Y. Ding , G.‐K. Xu , and G.‐F. Wang , “On the Determination of Elastic Moduli of Cells by AFM Based Indentation,” Scientific Reports 7 (2017): 45575. 28368053 10.1038/srep45575 PMC5377332

[38] T. D. Nguyen and Y. Gu , “Investigation of Cell‐Substrate Adhesion Properties of Living Chondrocyte by Measuring Adhesive Shear Force and Detachment Using AFM and Inverse FEA,” Scientific Reports 6 (2016): 38059. 27892536 10.1038/srep38059 PMC5125162

[39] N. Mittal , E. B. Michels , A. E. Massey , et al., “Myosin‐Independent Stiffness Sensing by Fibroblasts Is Regulated by the Viscoelasticity of Flowing Actin,” Communications Materials 5 (2024): 6. 38741699 10.1038/s43246-024-00444-0 PMC11090405

[40] M. H. Jo , J. Li , V. Jaumouillé , et al., “Single‐Molecule Characterization of Subtype‐Specific β1 Integrin Mechanics,” Nature Communications 13 (2022): 7471. 10.1038/s41467-022-35173-w PMC9719539 36463259

[41] S. ‐B. Han , G. Lee , D. Kim , et al., “Selective Suppression of Integrin‐Ligand Binding by Single Molecular Tension Probes Mediates Directional Cell Migration,” Advanced Science 11 (2024): 2306497. 38311584 10.1002/advs.202306497 PMC11005741

[42] X. Wang , Z. Rahil , I. T. S. Li , et al., “Constructing Modular and Universal Single Molecule Tension Sensor Using Protein G to Study Mechano‐Sensitive Receptors,” Scientific Reports 6 (2016): 21584. 26875524 10.1038/srep21584 PMC4753514

[43] Y. Murad and I. T. Li , “Quantifying Molecular Forces with Serially Connected Force Sensors,” Biophysical Journal 116 (2019): 1282–1291. 30902365 10.1016/j.bpj.2019.02.027 PMC6451047

[44] Y. Huang , C. Schell , T. B. Huber , et al., “Traction Force Microscopy with Optimized Regularization and Automated Bayesian Parameter Selection for Comparing Cells,” Scientific Reports 9 (2019): 539. 30679578 10.1038/s41598-018-36896-x PMC6345967

[45] C. Aermes , A. Hayn , T. Fischer , and C. T. Mierke , “Environmentally Controlled Magnetic Nano‐Tweezer for Living Cells and Extracellular Matrices,” Scientific Reports 10 (2020): 13453. 32778758 10.1038/s41598-020-70428-w PMC7417586

[46] M. Uhlen , C. Zhang , S. Lee , et al., “A Pathology Atlas of the Human Cancer Transcriptome,” Science 357 (2017): eaan2507. 28818916 10.1126/science.aan2507

[47] B. Kovacs , F. A. Kraft , Z. Szabo , Y. Nazirizadeh , M. Gerken , and R. Horvath , “Near Cut‐Off Wavelength Operation of Resonant Waveguide Grating Biosensors,” Scientific Reports 11 (2021): 13091. 34158570 10.1038/s41598-021-92327-4 PMC8219702

[48] V. S. Rajan , V. M. Laurent , C. Verdier , and A. Duperray , ”Unraveling the Receptor‐Ligand Interactions between Bladder Cancer Cells and the Endothelium Using AFM,“ Biophysical Journal 112 (2017): 1246–1257. 28355551 10.1016/j.bpj.2017.01.033 PMC5375142

[49] S. Cho , S. Rhee , C. M. Madl , et al., “Selective Inhibition of Stromal Mechanosensing Suppresses Cardiac Fibrosis,” Nature 642 (2025): 766–775. 40307543 10.1038/s41586-025-08945-9 PMC12176515

[50] V. A. Morikis , S. J. Chen , J. Madigan , et al., “β2‐Integrin Adhesive Bond Tension under Shear Stress Modulates Cytosolic Calcium Flux and Neutrophil Inflammatory Response,” Cells 11 (2022): 2822. 36139397 10.3390/cells11182822 PMC9497066

[51] Z. S. Wilson , H. Witt , L. Hazlett , et al., “Context‐Dependent Role of Vinculin in Neutrophil Adhesion, Motility and Trafficking,” Scientific Reports 10 (2020): 2142. 32034208 10.1038/s41598-020-58882-y PMC7005776

[52] Z. Zhang , Z. Zhao , X. Huang , et al., “Galectin‐3‐Integrin α5β1 Phase Separation Disrupted by Advanced Glycation End‐Products Impairs Diabetic Wound Healing in Rodents,” Nature Communications 16 (2025): 7287. 10.1038/s41467-025-62320-w PMC12332078 40775187

[53] S. Yuge , K. Nishiyama , Y. Arima , et al., “Mechanical Loading of Intraluminal Pressure Mediates Wound Angiogenesis by Regulating the TOCA Family of F‐BAR Proteins,” Nature Communications 13 (2022): 2594. 10.1038/s41467-022-30197-8 PMC9098626 35551172

[54] S. P. Wang , R. Chennupati , H. Kaur , A. Iring , N. Wettschureck , and S. Offermanns , “Endothelial Cation Channel PIEZO1 Controls Blood Pressure by Mediating Flow‐Induced ATP Release,” The Journal of Clinical Investigation 126 (2016): 4527–4536. 27797339 10.1172/JCI87343 PMC5127677

[55] D. De Vecchis , D. J. Beech , and A. C. Kalli , “Molecular Dynamics Simulations of Piezo1 Channel Opening by Increases in Membrane Tension,” Biophysical Journal 120 (2021): 1510–1521. 33582135 10.1016/j.bpj.2021.02.006 PMC8105709

[56] A. D.; Brugués , E. Anon , V. Conte , et al., “Forces Driving Epithelial Wound Healing,” Nature Physics 10 (2014): 683–690. 27340423 10.1038/nphys3040 PMC4915550

[57] A. Hayer , L. Shao , M. Chung , et al., “Engulfed Cadherin Fingers Are Polarized Junctional Structures between Collectively Migrating Endothelial Cells,” Nature Cell Biology 18 (2016): 1311–1323. 27842057 10.1038/ncb3438 PMC6159904

[58] S. Rahimi , W. V. J. Hariton , F. Khalaj , R. J. Ludwig , L. Borradori , and E. J. Müller , “Desmoglein‐Driven Dynamic Signaling in Pemphigus Vulgaris: A Systematic Review of Pathogenic Pathways,” npj Regenerative Medicine 10 (2025): 39. 40846710 10.1038/s41536-025-00426-x PMC12373888

[59] C. Bertocchi , Y. Wang , A. Ravasio , et al., “Nanoscale Architecture of Cadherin‐Based Cell Adhesions,” Nature Cell Biology 19 (2017): 28–37. 27992406 10.1038/ncb3456 PMC5421576

[60] D. Ollech , T. Pflästerer , A. Shellard , et al., “An Optochemical Tool for Light‐Induced Dissociation of Adherens Junctions to Control Mechanical Coupling between Cells,” Nature Communications 11 (2020): 472. 10.1038/s41467-020-14390-1 PMC6981158 31980653

[61] M. Gloerich , J. M. Bianchini , K. A. Siemers , D. J. Cohen , and W. J. Nelson , “Cell Division Orientation Is Coupled to Cell–cell Adhesion by the E‐Cadherin/LGN Complex,” Nature Communications 8 (2017): 2017. 10.1038/ncomms13996 PMC5216124 28045117

[62] N. Ishiyama , R. Sarpal , M. N. Wood , et al., “Force‐Dependent Allostery of the α‐Catenin Actin‐Binding Domain Controls Adherens Junction Dynamics and Functions,” Nature Communications 9 (2018): 5121. 10.1038/s41467-018-07481-7 PMC6269467 30504777

[63] G. R. Kale , X. Yang , J.‐M. Philippe , M. Mani , P.‐F. Lenne , and T. Lecuit , “Distinct Contributions of Tensile and Shear Stress on E‐Cadherin Levels during Morphogenesis,” Nature Communications 9 (2018): 5021. 10.1038/s41467-018-07448-8 PMC6258672 30479400

[64] N. Morales‐Camilo , J. Liu , M. J. Ramírez , et al., “Alternative Molecular Mechanisms for Force Transmission at Adherens Junctions via β‐Catenin‐Vinculin Interaction,” Nature Communications 15 (2024): 5608. 10.1038/s41467-024-49850-5 PMC11226457 38969637

[65] K. Duraivelan and D. Samanta , “Tracing the Evolution of Nectin and Nectin‐Like Cell Adhesion Molecules,” Scientific Reports 10, no. 1, 2020. 32523039 10.1038/s41598-020-66461-4 PMC7286890

[66] C. Offenhäuser , K. A. Dave , K. J. Beckett , et al., “EphA2 Regulates Vascular Permeability and Prostate Cancer Metastasis via Modulation of Cell Junction Protein Phosphorylation,” Oncogene 44 (2025): 208–227. 39511410 10.1038/s41388-024-03206-x PMC11753358

[67] W. T. Shelton , S. M. Thomas , H. R. Alexander , C. E. Thomes , D. E. Conway , and A. D. Dubash , “Desmoglein‐2 Harnesses a PDZ‐GEF2/Rap1 Signaling Axis to Control Cell Spreading and Focal Adhesions Independent of Cell–cell Adhesion,” Scientific Reports 11 (2021): 13295. 34168237 10.1038/s41598-021-92675-1 PMC8225821

[68] H. Ungewiß , F. Vielmuth , S. T. Suzuki , et al., “Desmoglein 2 Regulates the Intestinal Epithelial Barrier via p38 Mitogen‐Activated Protein Kinase,” Scientific Reports 7 (2017): 6329. 28740231 10.1038/s41598-017-06713-y PMC5524837

[69] F. Völlner , J. Ali , N. Kurrle , et al., “Loss of Flotillin Expression Results in Weakened Desmosomal Adhesion and Pemphigus Vulgaris‐Like Localisation of Desmoglein‐3 in Human Keratinocytes,” Scientific Reports 6 (2016): 28820. 27346727 10.1038/srep28820 PMC4922016

[70] C. Arbore , M. Sergides , L. Gardini , et al., “α‐Catenin Switches between a Slip and an Asymmetric Catch Bond with F‐Actin to Cooperatively Regulate Cell Junction Fluidity,” Nature Communications 13 (2022): 1146. 10.1038/s41467-022-28779-7 PMC8894357 35241656

[71] R. B. Troyanovsky , I. Indra , and S. M. Troyanovsky , “Actin‐Dependent α‐Catenin Oligomerization Contributes to Adherens Junction Assembly,” Nature Communications 16 (2025): 1801. 10.1038/s41467-025-57079-z PMC11842732 39979305

[72] M. Yao , W. Qiu , R. Liu , et al., “Force‐Dependent Conformational Switch of α‐Catenin Controls Vinculin Binding,” Nature Communications 5 (2014): 4525. 10.1038/ncomms5525 25077739

[73] Y. Campos , X. Qiu , E. Gomero , et al., “Alix‐Mediated Assembly of the Actomyosin–tight Junction Polarity Complex Preserves Epithelial Polarity and Epithelial Barrier,” Nature Communications 7 (2016): 11876. 10.1038/ncomms11876 PMC4931029 27336173

[74] Y. Ninoyu , H. Sakaguchi , C. Lin , et al., “The Integrity of Cochlear Hair Cells Is Established and Maintained through the Localization of Dia1 at Apical Junctional Complexes and Stereocilia,” Cell Death & Disease 11 (2020): 536. 32678080 10.1038/s41419-020-02743-z PMC7366933

[75] M. Maupérin , Y. Sun , T. Glandorf , et al., “A Feedback Circuitry Involving γ‐Actin, β‐Actin and Nonmuscle Myosin‐2 A Controls Tight Junction and Apical Cortex Mechanics,” Nature Communications 16 (2025): 2514. 10.1038/s41467-025-57428-y PMC11906862 40082413

[76] D. Pinheiro , E. Hannezo , S. Herszterg , et al., “Transmission of Cytokinesis Forces via E‐Cadherin Dilution and Actomyosin Flows,” Nature 545 (2017): 103–107. 28296858 10.1038/nature22041 PMC6143170

[77] E. McEvoy , T. Sneh , E. Moeendarbary , et al., “Feedback between Mechanosensitive Signaling and Active Forces Governs Endothelial Junction Integrity,” Nature Communications 13 (2022): 7089. 10.1038/s41467-022-34701-y PMC9675837 36402771

[78] P. Ringer , A. Weißl , A.‐L. Cost , et al., “Multiplexing Molecular Tension Sensors Reveals Piconewton Force Gradient across Talin‐1,” Nature Methods 14 (2017): 1090–1096. 28945706 10.1038/nmeth.4431

[79] M. A. K. Rätze , T. Koorman , T. Sijnesael , et al., “Loss of E‐Cadherin Leads to Id2‐Dependent Inhibition of Cell Cycle Progression in Metastatic Lobular Breast Cancer,” Oncogene 41 (2022): 2932–2944. 35437308 10.1038/s41388-022-02314-w PMC9122823

[80] Q. Tian , F. Yang , H. Jiang , et al., “Imaging and Detecting Intercellular Tensile Forces in Spheroids and Embryoid Bodies Using Lipid‐Modified DNA Probes,” Frontiers in Cell and Developmental Biology 11 (2023): 1220079. 37920824 10.3389/fcell.2023.1220079 PMC10619156

[81] T. Sadhanasatish , K. Augustin , L. Windgasse , A. Chrostek‐Grashoff , M. Rief , and C. Grashoff , “A Molecular Optomechanics Approach Reveals Functional Relevance of Force Transduction across Talin and Desmoplakin,” Science Advances 9 (2023): eadg3347. 37343090 10.1126/sciadv.adg3347 PMC10284548

[82] X. Jin , J. Rosenbohm , E. Kim , et al., “Desmosomal Cadherin Tension Loss in Pemphigus Vulgaris Mediated by the Inhibition of Active RhoA at Cell‐Cell Adhesions,” Iscience 28 (2025): 113081. 40740498 10.1016/j.isci.2025.113081 PMC12307676

[83] A. Labernadie , T. Kato , A. D.; Brugués , et al., “A Mechanically Active Heterotypic E‐Cadherin/N‐Cadherin Adhesion Enables Fibroblasts to Drive Cancer Cell Invasion,” Nature Cell Biology 19 (2017): 224–237. 28218910 10.1038/ncb3478 PMC5831988

[84] A. Weber , M. D. Vivanco , and J. L. Toca‐Herrera , “Application of Self‐Organizing Maps to AFM‐Based Viscoelastic Characterization of Breast Cancer Cell Mechanics,” Scientific Reports 13 (2023): 3087. 36813800 10.1038/s41598-023-30156-3 PMC9947176

[85] M. J. White , M. Ozkan , J. E. G. Medellin , A. Solanki , and J. A. Hubbell , “Inhibition of Talin2 Dedifferentiates Myofibroblasts and Reverses Lung and Kidney Fibrosis,” Scientific Reports 15 (2025): 18010. 40410300 10.1038/s41598-025-00939-x PMC12102334

[86] N. Braidotti , G. Demontis , M. Conti , et al., “The Local Mechanosensitive Response of Primary Cardiac Fibroblasts Is Influenced by the Microenvironment Mechanics,” Scientific Reports 14 (2024): 10365. 38710778 10.1038/s41598-024-60685-4 PMC11074268

[87] Y. Ma , J. Yue , Y. Zhang , et al., “ACF7 Regulates Inflammatory Colitis and Intestinal Wound Response by Orchestrating Tight Junction Dynamics,” Nature Communications 8 (2017): 15375. 10.1038/ncomms15375 PMC5458510 28541346

[88] O. Ilina , P. G. Gritsenko , S. Syga , et al., “Cell–cell Adhesion and 3D Matrix Confinement Determine Jamming Transitions in Breast Cancer Invasion,” Nature Cell Biology 22 (2020): 1103–1115. 32839548 10.1038/s41556-020-0552-6 PMC7502685

[89] Y. Koike , M. Yozaki , A. Utani , and H. Murota , “Fibroblast Growth Factor 2 accelerates the Epithelial–mesenchymal Transition in Keratinocytes during Wound Healing Process,” Scientific Reports 10 (2020): 18545. 33122782 10.1038/s41598-020-75584-7 PMC7596476

[90] E. Bazellières , V. Conte , A. Elosegui‐Artola , et al., “Control of Cell–cell Forces and Collective Cell Dynamics by the Intercellular Adhesome,” Nature Cell Biology 17 (2015): 409–420. 25812522 10.1038/ncb3135 PMC4886824

[91] M. Dieding , J. D. Debus , R. Kerkhoff , et al., “Arrhythmogenic Cardiomyopathy Related DSG2 Mutations Affect Desmosomal Cadherin Binding Kinetics,” Scientific Reports 7 (2017): 13791. 29062102 10.1038/s41598-017-13737-x PMC5653825

[92] A. M. Sigmund , M. Winkler , S. Engelmayer , et al., “Apremilast Prevents Blistering in Human Epidermis and Stabilizes Keratinocyte Adhesion in Pemphigus,” Nature Communications 14 (2023): 116. 10.1038/s41467-022-35741-0 PMC9829900 36624106

[93] A. M. Sigmund , L. S. Steinert , D. T. Egu , F. C. Bayerbach , J. Waschke , and F. Vielmuth , “Dsg2 Upregulation as a Rescue Mechanism in Pemphigus,” Frontiers in Immunology 11 (2020): 581370. 33193387 10.3389/fimmu.2020.581370 PMC7655986

[94] V. R. Singh , Y. A. Yang , H. Yu , R. D. Kamm , Z. Yaqoob , and P. T. C. So , “Studying Nucleic Envelope and Plasma Membrane Mechanics of Eukaryotic Cells Using Confocal Reflectance Interferometric Microscopy,” Nature Communications 10 (2019): 3652. 10.1038/s41467-019-11645-4 PMC6692322 31409824

[95] R. Taniguchi , C. Orniacki , J. P. Kreysing , et al., “Nuclear Pores Safeguard the Integrity of the Nuclear Envelope,” Nature Cell Biology 27 (2025): 7062–775. 10.1038/s41556-025-01648-3 PMC12081302 40205196

[96] I. Andreu , I. Granero‐Moya , N. R. Chahare , et al., “Mechanical Force Application to the Nucleus Regulates Nucleocytoplasmic Transport,” Nature Cell Biology 24 (2022): 896–905. 35681009 10.1038/s41556-022-00927-7 PMC7614780

[97] Y. Sakiyama , A. Mazur , L. E. Kapinos , and R. Y. Lim , “Spatiotemporal Dynamics of the Nuclear Pore Complex Transport Barrier Resolved by High‐Speed Atomic Force Microscopy,” Nature Nanotechnology 11 (2016): 719–723. 10.1038/nnano.2016.62 27136131

[98] S. Mosalaganti , J. Kosinski , S. Albert , et al., “In Situ Architecture of the Algal Nuclear Pore Complex,” Nature Communications 9 (2018): 2361. 10.1038/s41467-018-04739-y PMC6006428 29915221

[99] P. Nastały , D. Purushothaman , S. Marchesi , et al., “Role of the Nuclear Membrane Protein Emerin in Front‐Rear Polarity of the Nucleus,” Nature Communications 11 (2020): 2122. 10.1038/s41467-020-15910-9 PMC7195445 32358486

[100] A. J. Earle , T. J. Kirby , G. R. Fedorchak , et al., “Mutant Lamins cause Nuclear Envelope Rupture and DNA Damage in Skeletal Muscle Cells,” Nature Materials 19 (2020): 464–473. 31844279 10.1038/s41563-019-0563-5 PMC7102937

[101] K. T. Sapra , Z. Qin , A. Dubrovsky‐Gaupp , et al., “Nonlinear Mechanics of Lamin Filaments and the Meshwork Topology Build an Emergent Nuclear Lamina,” Nature Communications 11 (2020): 6205. 10.1038/s41467-020-20049-8 PMC7718915 33277502

[102] K. V. Iyer , A. Taubenberger , S. A. Zeidan , N. A. Dye , S. Eaton , and F. Jülicher , “Apico‐Basal Cell Compression Regulates Lamin A/C Levels in Epithelial Tissues,” Nature Communications 12 (2021): 1756. 10.1038/s41467-021-22010-9 PMC7994818 33767161

[103] T. O. Ihalainen , L. Aires , F. A. Herzog , R. Schwartlander , J. Moeller , and V. Vogel , “Differential Basal‐to‐Apical Accessibility of Lamin A/C Epitopes in the Nuclear Lamina Regulated by Changes in Cytoskeletal Tension,” Nature Materials 14 (2015): 1252–1261. 26301768 10.1038/nmat4389 PMC4655446

[104] R. M. Skory , A. A. Moverley , G. Ardestani , et al., “The Nuclear Lamina Couples Mechanical Forces to Cell Fate in the Preimplantation Embryo via Actin Organization,” Nature Communications 14 (2023): 3101. 10.1038/s41467-023-38770-5 PMC10226985 37248263

[105] M. Dhankhar , Z. Guo , A. Kant , et al., “Revealing the Biophysics of Lamina‐Associated Domain Formation by Integrating Theoretical Modeling and High‐Resolution Imaging,” Nature Communications 16 (2025): 7909. 10.1038/s41467-025-63244-1 PMC12378204 40854894

[106] J. A. Jackson , N. Romeo , A. Mietke , et al., “Scaling Behaviour and Control of Nuclear Wrinkling,” Nature Physics 19 (2023): 1927–1935. 38831923 PMC11146749

[107] B. D. Cosgrove , C. Loebel , T. P. Driscoll , et al., “Nuclear Envelope Wrinkling Predicts Mesenchymal Progenitor Cell Mechano‐Response in 2D and 3D Microenvironments,” Biomaterials 270 (2021): 120662. 33540172 10.1016/j.biomaterials.2021.120662 PMC7936657

[108] T.‐C. Wang , S. Abolghasemzade , B. P. McKee , et al., “Matrix Stiffness Drives Drop like Nuclear Deformation and Lamin A/C Tension‐Dependent YAP Nuclear Localization,” Nature Communications 15 (2024): 10151. 10.1038/s41467-024-54577-4 PMC11584751 39578439

[109] T.‐C. Wang , C. R. Dollahon , S. Mishra , et al., “Extreme Wrinkling of the Nuclear Lamina Is a Morphological Marker of Cancer,” npj Precision Oncology 8 (2024): 276. 39623008 10.1038/s41698-024-00775-8 PMC11612457

[110] A. J. Lomakin , C. J. Cattin , D. Cuvelier , et al., “The Nucleus Acts as a Ruler Tailoring Cell Responses to Spatial Constraints,” Science 370 (2020): eaba2894. 33060332 10.1126/science.aba2894 PMC8059074

[111] R. J. Chai , H. Werner , P. Y. Li , et al., “Disrupting the LINC Complex by AAV Mediated Gene Transduction Prevents Progression of Lamin Induced Cardiomyopathy,” Nature Communications 12 (2021): 4722. 10.1038/s41467-021-24849-4 PMC8342462 34354059

[112] S. G. Alam , Q. Zhang , N. Prasad , et al., “The Mammalian LINC Complex Regulates Genome Transcriptional Responses to Substrate Rigidity,” Scientific Reports 6 (2016): 38063. 27905489 10.1038/srep38063 PMC5131312

[113] M. Hieda , T. Matsumoto , M. Isobe , et al., “The SUN2‐Nesprin‐2 LINC Complex and KIF20A Function in the Golgi Dispersal,” Scientific Reports 11 (2021): 5358. 33686165 10.1038/s41598-021-84750-4 PMC7940470

[114] J.‐E. Park , J. Jo , K. Xu , et al., “Attenuated Nuclear Tension Regulates Progerin‐Induced Mechanosensitive Nuclear Wrinkling and Chromatin Remodeling,” Advanced Science 12 (2025): 2502375. 40344643 10.1002/advs.202502375 PMC12376529

[115] P. T. Arsenovic , I. Ramachandran , K. Bathula , et al., “Nesprin‐2G, a Component of the Nuclear LINC Complex, Is Subject to Myosin‐Dependent Tension,” Biophysical Journal 110 (2016): 34–43. 26745407 10.1016/j.bpj.2015.11.014 PMC4805861

[116] Y. Wu , Y. Song , J. Soto , et al., “Viscoelastic Extracellular Matrix Enhances Epigenetic Remodeling and Cellular Plasticity,” Nature Communications 16 (2025): 4054. 10.1038/s41467-025-59190-7 PMC12043949 40307238

[117] M. M. Nava , Y. A. Miroshnikova , L. C. Biggs , et al., “Heterochromatin‐Driven Nuclear Softening Protects the Genome against Mechanical Stress‐Induced Damage,” Cell 181, 800–817.e22. 32302590 10.1016/j.cell.2020.03.052 PMC7237863

[118] B. Wang , R. Kronenberg‐Tenga , V. Rosti , et al., “The Molecular Basis of Lamin‐Specific Chromatin Interactions,” Nature Structural & Molecular Biology 32 (2025): 1999–2011. 10.1038/s41594-025-01622-5 PMC12527912 40750945

[119] I. Bronshtein , E. Kepten , I. Kanter , et al., “Loss of Lamin A Function Increases Chromatin Dynamics in the Nuclear Interior,” Nature Communications 6 (2015): 8044. 10.1038/ncomms9044 PMC4560783 26299252

[120] G. Bergamaschi , A. S. Biebricher , H. Witt , et al., “Heterogeneous Force Response of Chromatin in Isolated Nuclei,” Cell Reports 43 (2024): 114852. 39412986 10.1016/j.celrep.2024.114852

[121] A. Mitra , M. F. A. Cutiongco , R. Burla , et al., “Acute Chromatin Decompaction Stiffens the Nucleus as Revealed by Nanopillar‐Induced Nuclear Deformation in Cells,” Proceedings of the National Academy of Sciences 122 (2025): e2416659122. 10.1073/pnas.2416659122 PMC12088434 40343993

[122] Q. Zhang , A. C. Tamashunas , and T. P. Lele , “A Direct Force Probe for Measuring Mechanical Integration between the Nucleus and the Cytoskeleton,” Journal of Visualized Experiments 137 (2018): 58038. 10.3791/58038 PMC6092038 30102282

[123] A. Kaczmarczyk , H. Meng , O. Ordu , J.v Noort , and N. H. Dekker , “Chromatin Fibers Stabilize Nucleosomes under Torsional Stress,” Nature Communications 11 (2020): 126. 10.1038/s41467-019-13891-y PMC6949304 31913285

[124] S. Hu , D. J. Chapski , N. D. Gehred , et al., “Histone H1. 0 Couples Cellular Mechanical Behaviors to Chromatin Structure,” Nature Cardiovascular Research 3 (2024): 441–459. 10.1038/s44161-024-00460-w PMC11101354 38765203

[125] A. D. Stephens , E. J. Banigan , S. A. Adam , R. D. Goldman , and J. F. Marko , ”Chromatin and Lamin A Determine Two Different Mechanical Response Regimes of the Cell Nucleus,“ Molecular Biology of the Cell 28 (2017): 1984–1996. 28057760 10.1091/mbc.E16-09-0653 PMC5541848

[126] Z. Dou , K. Ghosh , M. G. Vizioli , et al., “Cytoplasmic Chromatin Triggers Inflammation in Senescence and Cancer,” Nature 550 (2017): 402–406. 28976970 10.1038/nature24050 PMC5850938

[127] J. L. Mehl , A. Earle , J. Lammerding , M. Mhlanga , V. Vogel , and N. Jain , “Blockage of Lamin‐A/C Loss Diminishes the pro‐Inflammatory Macrophage Response,” Iscience 25 (2022): 105528. 36465100 10.1016/j.isci.2022.105528 PMC9708799

[128] F. J. Ferreira , M. Galhardo , J. M. Nogueira , J. Teixeira , E. Logarinho , and J. Bessa , “FOXM1 Expression Reverts Aging Chromatin Profiles through Repression of the Senescence‐Associated Pioneer Factor AP‐1,” Nature Communications 16 (2025): 2931. 10.1038/s41467-025-57503-4 PMC11937471 40133272

[129] J. Perovanovic , S. Dell’Orso , V. F. Gnochi , et al., “Laminopathies Disrupt Epigenomic Developmental Programs and Cell Fate,” Science Translational Medicine 8 (2016): 335ra358–335ra358. 10.1126/scitranslmed.aad4991 PMC4939618 27099177

[130] Y. Wang , A. Elsherbiny , L. Kessler , et al., “Lamin A/C‐Dependent Chromatin Architecture Safeguards Naïve Pluripotency to Prevent Aberrant Cardiovascular Cell Fate and Function,” Nature Communications 13 (2022): 6663. 10.1038/s41467-022-34366-7 PMC9636150 36333314

